# Optimization of light exposure and sleep schedule for circadian rhythm entrainment

**DOI:** 10.1371/journal.pone.0251478

**Published:** 2021-06-08

**Authors:** Jiawei Yin, A. Agung Julius, John T. Wen

**Affiliations:** 1 Institute of Oceanographic Instrumentation, Qilu University of Technology (Shandong Academy of Sciences), Qingdao, China; 2 Department of Electrical, Computer, and Systems Engineering, Rensselaer Polytechnic Institute, Troy, New York, United States of America; 3 Lighting Enabled Systems and Applications (LESA) Engineering Research Center, Rensselaer Polytechnic Institute, Troy, New York, United States of America; Sichuan University, CHINA

## Abstract

The circadian rhythm, called Process C, regulates a wide range of biological processes in humans including sleep, metabolism, body temperature, and hormone secretion. Light is the dominant synchronizer of the circadian rhythm—it has been used to regulate the circadian phase to cope with jet-lag, shift work, and sleep disorder. The homeostatic oscillation of the sleep drive is called Process S. Process C and Process S together determine the sleep-wake cycle in what is known as the two-process model. This paper addresses the regulation of *both* Process C and Process S by scheduling light exposure and sleep based on numerical simulations of circadian rhythm and sleep mathematical models. This is a significant step beyond the existing literature that only considers the entrainment of Process C. Regulation of the two-process model poses several unique features and challenges: 1. Process S is non-smooth, i.e., the homeostatic dynamics are different in the sleep and wake regimes; 2. Light only indirectly affects Process S through Process C; 3. Light does not affect Process C during sleep. We consider two scenarios: optimizing light intensity as the control input with spontaneous (i.e., unscheduled) sleep/wake times and jointly optimizing the light intensity and the sleep/wake times, which allows limited delayed sleep and early waking as part of the decision variables. We solve the time-optimal entrainment problem for the two-process model for both scenarios using an extension of the gradient descent algorithm to non-smooth systems. To illustrate the efficacy of our time-optimal entrainment strategies, we consider two common use cases: transmeridian travelers and shift workers. For transmeridian travelers, joint optimization of the two-process model avoids the unrealistic long wake duration when only Process C is considered. The entrainment time also decreases when both the light input and the sleep schedule are optimized compared to when only the light input is optimized. For shift workers, we show that the entrainment time is significantly shortened by optimizing the night shift working light.

## 1 Introduction

Sleep plays a significant role in our lives as it allows the body to recuperate and regenerate cells. It is tied to neurobehavioral performance: sleep deprivation and disorder have been empirically linked to degeneration of neurocognitive function [1, 2] and disruption of the circadian rhythm [3, 4]. The sleep-wake cycle is modeled with two separate processes: Process S models the sleep drive generated by the accumulation of sleep-inducing substances in the brain and Process C represents the sleep pressure generated by circadian rhythm [5, 6]. Process S may be modeled by the exponential growth and decay of the sleep drive during the waking and sleep periods [7], based on the similar pattern of the EEG power. Light (in the blue spectrum) is the dominant influence on Process C, though other factors such as exercise and meals also play a role. Empirical nonlinear forced oscillator models have been proposed for Process C based on the oscillation of circadian rhythm markers such as the core body temperature [8–10]. Experimental results in [6, 11] have demonstrated that the spontaneous sleep/wake times are jointly determined by Process S and Process C.

As light is the dominant synchronizer of the circadian rhythm, it has been used for regulating the circadian rhythm against issues such as jet-lag, shift work, and sleep disorder. Such a problem may be posed as a minimum-time optimal control problem: finding the lighting schedule to drive the circadian rhythm to a reference pattern in the shortest time possible, subject to the light intensity constraints. In [12], the problem is solved with a switching light input (bang-off control) for the nonlinear oscillator model in [10]. The time points between the light-on and light-off intervals are updated with a gradient descent algorithm. In [13], an optimal light control strategy has been proposed for the Drosophila circadian phase alignment using a reduced order phase dynamics model. Such formulation has been extended to phase synchronization of complex oscillator networks [14]. In our earlier work [15], we have used the Pontryagin Minimum Principle to efficiently compute the optimal light input for both the nonlinear oscillator model and phase reduced model.

In this paper, we solve a larger problem beyond the current literature of light-based circadian rhythm entrainment: how to entrain both Process C and Process S using the light input, and possibly the sleep scheduling. We first consider the spontaneous sleep/wake case, with the light intensity as the input. We then include the sleep/wake times as additional input variables. The combined circadian rhythm and sleep entrainment problem poses several challenges: 1. Process S is non-smooth, as the homeostatic dynamics depend on the sleep state; 2. Light input only indirectly affects sleep, through circadian rhythm; 3. Light input is decoupled from the system during the sleep phase. To address these challenges, we apply optimal control for a hybrid dynamical system that contains two modes: sleep and wake. In [16], the problem is solved using optimization of the mode-switching points. We apply variational calculus to evaluate the co-state discontinuity around the switching times and use the gradient descent algorithm to determine the optimal light intensity. In [17], we report our initial results for the spontaneous sleep/wake case. Here we include sleep scheduling as additional input variables. As in our work on Process C regulation [15], several reduced order models are also considered to understand the impact of model reduction on the optimal entrainment time. To reduce the dependence on model parameters, we use the optimal control solution to train a feedback controller that adjusts the light intensity and sleep schedule based on the states of Process C and Process S. To our knowledge, our work is the first optimal control results involving the combined Process C and Process S dynamics.

To illustrate the application of the optimal entrainment algorithms, we consider two common but challenging cases: jet-lag adjustment for travelers and recovery of shift workers. For transmeridian travelers with only Process C considered, the optimal lighting requires excessive waking time (longer than 20 hours in some cases) which leads to suppressed amplitude of the circadian oscillation [12, 15]. When Process S is included in the optimization, such long waking times and amplitude suppression are avoided. The optimal Process C entrainment also exhibits an east-west symmetry, i.e., the entrainment times are almost identical for the same amount of delay (going west) or advance (going east) [12, 15]. This is no longer true with the introduction of Process S. The optimal solution for the combined processes shows a preference towards delay, i.e., longer entrainment times traveling eastward. This observation agrees previously reported results in [18], where the authors consider entrainment of Process C under periodic light cycles, and in [19], where the authors consider the entrainment of a network of the brain’s Suprachiasmatic Nuclei (SCN) also under periodic light cycles. Note that in both forementioned papers, the authors do not consider optimal entrainment or the sleep-wake process. Further, the addition of sleep scheduling significantly reduces the impact of jet-lag, e.g., for the 12-hour shift case, the entrainment time is decreased by 27% by optimizing both light and sleep schedule. For night shift workers, the optimal control solution shows that optimization of light input and wearing goggles to block part of the blue spectrum during shift work result in significantly lower entrainment time to return to the regular schedule.

## 2 Two-process modeling approach

Circadian rhythm regulation is performed under the dynamics of the circadian rhythm with the sleep homeostatic process. These two processes together form a model of the sleep-wake cycle, called the *two-process model*. The mathematical circadian rhythm model used in this paper is the Jewett-Forger-Kronauer (JFK) model in [12], which is a 3rd-order nonlinear differential equation and formulated based on the dynamics of the core body temperature. We will also discuss a 2nd-order circadian rhythm model and a 1st-order phase-reduced model to show the impacts of model reduction on the entrainment time and optimal solution. We model the sleep homeostatic process and sleep-wake cycle using Achermann’s two-process model [20]. Note that the circadian rhythm of the two-process model in [20] has no dynamics; it is represented as a pre-determined skewed sine wave that ignores the lighting impact. Therefore, this model has not been studied for light-based minimum-time entrainment. The JFK model in [12] has been updated from previous models to fit the latest empirical data and incorporate the lighting impacts on core body temperature. The minimum-time entrainment problem of this model has been studied in [12, 15]. However, this model, as well as the corresponding entrainment studies, ignores the sleep-wake cycle and sleep homeostatic process. As shown in Table 1, we formulate a two-process model in this paper by combining the JFK model with the homeostatic process and study the minimum-time entrainment based on this model.

**Table 1:** Modeling framework.

Model	Sleep dynamics	Updated circadian rhythm model	Minimum-time entrainment
Two-process model [20]	Yes	No	No
JFK model [12]	No	Yes	Yes (in [12, 15])
Models by Booth et al [21–23]	Yes (with NREM and REM)	No	No
Our two-process model	Yes	Yes	Yes

We note that there have been earlier work that provided more comprehensive models of the sleep-wake dynamic, e.g., those that consider rapid eye movement (REM) and non-rapid eye movement (NREM) sleep stages. Diniz Behn and Booth [21] proposed a two-time scale model consisting of fast synaptic-based interactions between neuronal populations and slow homeostatic and circadian processes for the dynamics of REM sleep. Later, they used the same mathematical tool (fast–slow decomposition) to analyze several physiologically-based mathematical models of sleep–wake and sleep stage regulation [22]. In this paper, we primarily focus on the daily dynamics of the sleep-wake process and the entrainment of these circadian and sleep-wake rhythms. Therefore, we choose to use the two-process model for our study.

### 2.1 Mathematical models for Process C

#### 2.1.1 Full (third-order) JFK circadian rhythm model

Based on the whole process through which light stimuli work on the hypothalamus and affect the core body temperature (CBT) [9], the JFK circadian rhythm model in [12] is separated into two parts: the first part is a light processor that models the transduction process on the retina, which transfers light energy into neuron stimulus. This part is called *Process L* and its dynamics are expressed as a 1st-order ODE, given as Eq (1a)–(1c):

$$\begin{array}{c} \begin{array}{cc} \frac{dn}{dt} & =60(\alpha (I)(1-n)-\gamma n), \end{array} \end{array}$$ (1a)

$$\begin{array}{c} \begin{array}{cc} \alpha (I) & =\alpha_{0}\cdot {\left(\frac{I}{I_{0}}\right)}^{p}, \end{array} \end{array}$$ (1b)

$$\begin{array}{c} \begin{array}{cc} u & =G\alpha (I)(1-n), \end{array} \end{array}$$ (1c)

where the parameter values are set as *α*_0_ = 0.05 h^−1^, *γ* = 0.0075 h^−1^, *I*_0_ = 9500 lux, *G* = 33.75, *p* = 0.5. The unit of time *t* in this paper is hour (h). Light intensity is denoted by *I* in lux. The variable *u* represents the circadian drive that works on the hypothalamus. The terms *n* and 1 − *n* represent the fractions of retinal cells in used state and ready state, respectively. Used state means the retinal cells have been stimulated by light and emitted action potentials, while ready state means the retinal cells are ready to accept the light signal and generate a neuron stimulus. The second part of the model simulates the whole process where the neuron stimulus works on the hypothalamus and affects the running of the core body temperature, which is called *Process P* in the JFK model. This part is expressed as the 2nd-order nonlinear differential equation:

$$\begin{array}{cc} \frac{dx}{dt} & =\frac{\pi }{12}[x_{c}+\mu (\frac{1}{3}x+\frac{4}{3}x^{3}-\frac{256}{105}x^{7})+\left(1-0.4x\right)\left(1-k_{c}x_{c}\right)u] \end{array}$$ (2a)

$$\begin{array}{cc} \frac{dx_{c}}{dt} & =\frac{\pi }{12}[\left(qx_{c}-kx\right)\left(1-0.4x\right)\left(1-k_{c}x_{c}\right)u-{\left(\frac{24}{0.99729\tau_{x}}\right)}^{2}x]. \end{array}$$ (2b)

Here *x*(*t*) is the state of core body temperature, *x*_*c*_(*t*) is a complementary state with a unit of h^−1^. The circadian drive *u*(*t*) is the input of the core body temperature oscillator, downstream from Process L. The values of the parameters in this part are given as *μ* = 0.13 h^−1^, *q* = 1/3, *τ*_*x*_ = 24.2 h, *k* = 0.55 h^−1^, *k*_*c*_ = 0.4 h.

We should note that the JFK model was formulated to simulate the dynamics of core body temperature under white light [8, 9]. Previous studies suggest that the melatonin secretion suppression [24, 25], circadian gene expression [26], sleepiness, and alertness [27] demonstrate spectral sensitivity to light, i.e., they are more sensitive to short-wavelength light. We represent the circadian spectral sensitivity to light wavelength as *R*(λ), where λ is the light wavelength. If experimental data about light spectral sensitivity of humans’ core body temperature are available in the future, we can formulate the term *R*(λ) and incorporate it into the JFK model by rewriting the Eq (1b) in Process L as

$$\begin{array}{c} \alpha \left(I\right)=\alpha_{0}\cdot {\left[\frac{\int R(\lambda )\cdot I(\lambda )d\lambda }{I_{0}}\right]}^{p}, \end{array}$$

where *I*(λ) represents the spectral density of the light intensity. The experimental studies in [28, 29] demonstrated that the melatonin, cortisol, circadian gene (*per2* and *Bmal1* mRNA), and subjective alertness of subjects staying under the filtered fluorescent light (filtering short wavelength spectrum less than 480 nm) are similar to those of subjects staying under darkness. Therefore, *R*(λ) ≈ 0 for λ > 480 nm.

In the subsequent subsections, we review two reduced-order versions of the full JFK model. The purpose of including these models is to evaluate whether optimal entrainment strategies that are computed for these models can be effectively used on the full-order model.

#### 2.1.2 Second-order circadian rhythm model

We observe from the Process L model in Eq (1a)–(1c) that when the light is off, *I*(*t*) = 0 and $\frac{dn}{dt}<0$, the used state transfers to the ready state and *n* gradually converges to 0. When the light is on, the light input drives the ready state to used state gradually. The dynamics of *n* reach an equilibrium if *α*(*I*)(1 − *n*) = *γn*, *u*(*t*) and *n*(*t*) also reach steady states with values given as

$$\begin{array}{c} n=\frac{\alpha (I)}{\alpha (I)+\gamma },\;u=G\frac{\gamma \alpha (I)}{\alpha (I)+\gamma }. \end{array}$$ (3)

**Fig 1:**
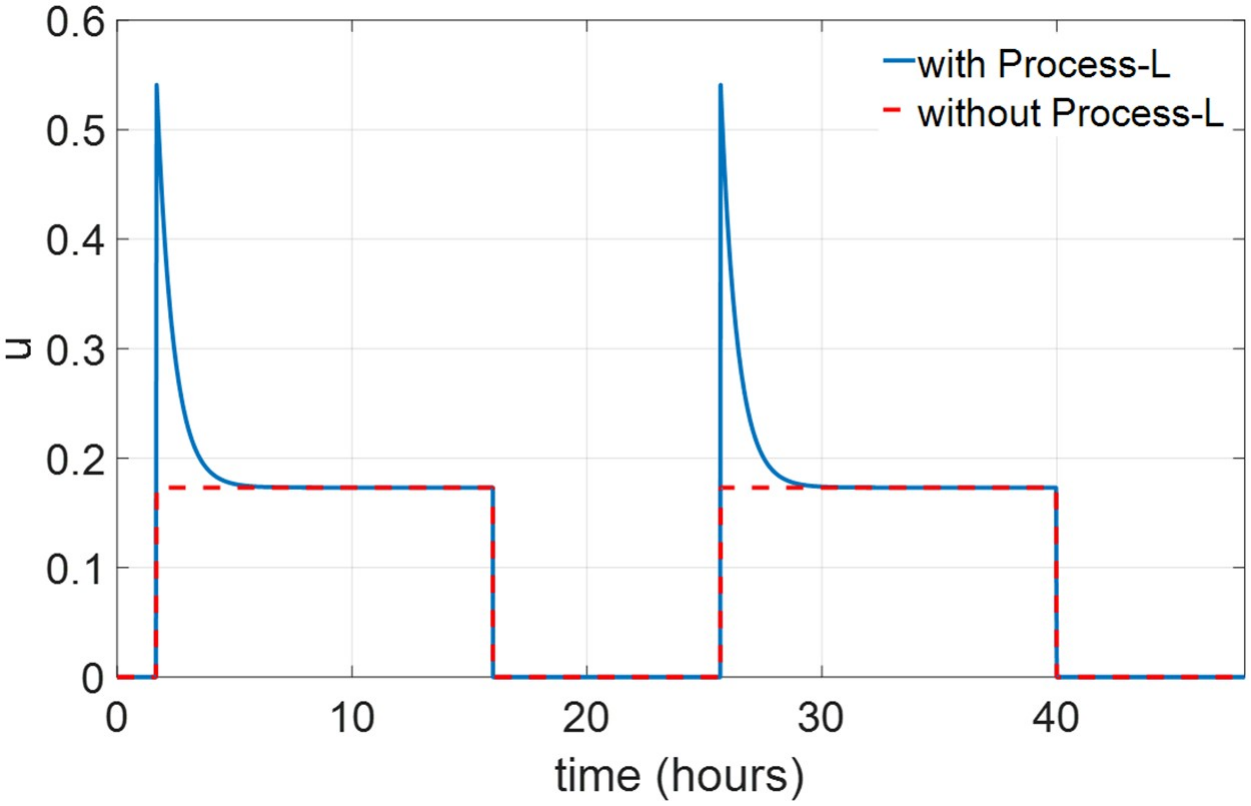
Circadian drive *u* with and without Process L.

The dynamics of the state *n* in Process L have a fast time scale compared to that of the circadian oscillation. We assume that Process L is always at its steady state, and the values of *n* and *u* are given as (3) when the light is on. Fig 1 compares the circadian drives *u*(*t*) with and without consideration of Process L. Note that *u*(*t*) reaches a peak value as a result of the transient response of *n* in Process L when the light is turned on, then sharply drops to the steady state value. After we ignore the transient response, the circadian drive is given as the red dash curve in Fig 1. This model only contains two states [*x*, *x*_*c*_]^*T*^ with dynamics given in Eq (2a) and (2b). The circadian drive *u*(*t*) is treated as the input of the 2nd-order circadian rhythm model.

#### 2.1.3 First-order circadian phase reduced model

**Phase response function** is widely used in the studies of circadian rhythms [30]. This function defines the effect of a light pulse on a circadian rhythm and reduces a high-order model into a one-dimensional phase-based model. In this paper, we define the circadian phase *θ* based on Process P states as

$$\begin{array}{c} \theta \overset{\Delta }{=}\{\begin{array}{cc} \text{arctan}(\frac{-x_{c}}{x}), & \text{if}\;x\geq 0,\\ \text{arctan}(\frac{-x_{c}}{x})+\pi , & \text{if}\;x<0, \end{array}\theta \in \left[0,2\pi \right), \end{array}$$ (4)

where *θ* is expressed in rad. The dynamics of the circadian phase are given as

$$\begin{array}{c} \frac{d\theta }{dt}=\omega_{0}+f\left(\theta \right)u\left(t\right), \end{array}$$ (5)

where *ω*_0_ is the so-called *free-running frequency*, expressed in rad/h. The state and phase without light input (*u* ≡ 0) are called the *free-running state* and *free-running phase*, respectively. The free-running period of the core body temperature oscillator is about 24.2 hours [9]. Therefore, we set *ω*_0_ = 2*π*/24.2 rad/h. The function *f*(*θ*) is the *phase response function* or *phase response curve (PRC)* of the circadian rhythm model. Four different methods for the measurement of a phase response function were summarized in [31]. In this paper, the phase response curve *f*(*θ*) is estimated by applying a 30-min light pulse with intensity *u* = 0.1731 (corresponding to *I* = 1000 lux) on the 2nd-order model in Section 2.1.2 and calculating the difference between the circadian phase having been stimulated by the light pulse and the free-running phase [15].

### 2.2 Mathematical model for Process S

The sleep homeostasis *H*(*t*) represents the accumulation of a substance that generates the sleep drive. It works like an internal timer that describes the tendency to fall asleep if the subject is awake and the tendency to wake up if the subject is asleep. The dynamics of *H*(*t*) during sleep and wake cycles are modeled respectively as

$$\begin{array}{c} \frac{dH}{dt}=\{\begin{array}{cc} -H/\tau_{d}, & \beta (t)=1,\\ \left(1-H\right)/\tau_{r}, & \beta (t)=0. \end{array} \end{array}$$ (6)

The variable *β*(*t*) ∈ {0, 1} indicates whether the subject is asleep (*β*(*t*) = 1) or awake (*β*(*t*) = 0) at time *t*. Eq (6) implies *H*(*t*) → 0 as *t* → ∞ when *β* ≡ 1 and *H*(*t*)→1 as *t* → ∞ when *β* ≡ 0. The parameters *τ*_*r*_ = 18.2 h and *τ*_*d*_ = 4.2 h define the time scales of these dynamics, which are estimated based on experimental data from a study with 8 healthy human subjects [32]. These parameters are within the standard deviation of the parameters reported in a more recent study in [33].

### 2.3 Two-process model

Sleepiness, denoted as *B*(*t*), quantitatively represents a subject’s tendency to fall asleep. It is affected by both the sleep homeostasis and the circadian state. The work in [34] defined the value of sleepiness by the JFK circadian rhythm model in Section 2.1.1 and sleep homeostasis as

$$\begin{array}{c} B=H-A_{c}x, \end{array}$$ (7)

and showed that the values of sleepiness predicted by the model were similar to the empirical data, where *A*_*c*_ = 0.1333. If Process C is represented by the 2nd-order model in Section 2.1.2, the sleepiness value is still defined by Eq (7). When we use the phase reduced model in Section 2.1.3 to represent Process C, based on the approximation that the periodic orbit of the JFK model is very close to a unit circle [15], we have

x≈cos(θ).

The sleepiness value of the circadian phase-sleep two-process model is given as

$$\begin{array}{c} B=H-A_{c}\cos\left(\theta \right). \end{array}$$ (8)

This model can be further generalized by replacing *A*_*c*_cos(*θ*) with other 2*π*-periodic functions of *θ* [34]. Based on the model presented in [20], if a subject follows the spontaneous sleep schedule, the sleep state *β*(*t*) is completely determined by the value of *B* and the sleep state in the previous time. When *B* is equal to an upper threshold of *H*_*m*_ = 0.67, the subject feels tired enough to sleep. The subject wakes up spontaneously when *B* reaches a lower threshold of *L*_*m*_ = 0.17. The spontaneous wake time *T*_wake_ and sleep time *T*_sleep_ are defined as

$$\begin{array}{c} \left\{ \begin{array}{c} T_{\text{wake}}=\left\{t\;|\;B\left(t\right)=L_{m}=0.17\right\},\\ T_{\text{sleep}}=\left\{t\;|\;B\left(t\right)=H_{m}=0.67\right\}. \end{array}\right. \end{array}$$

**Fig 2:**
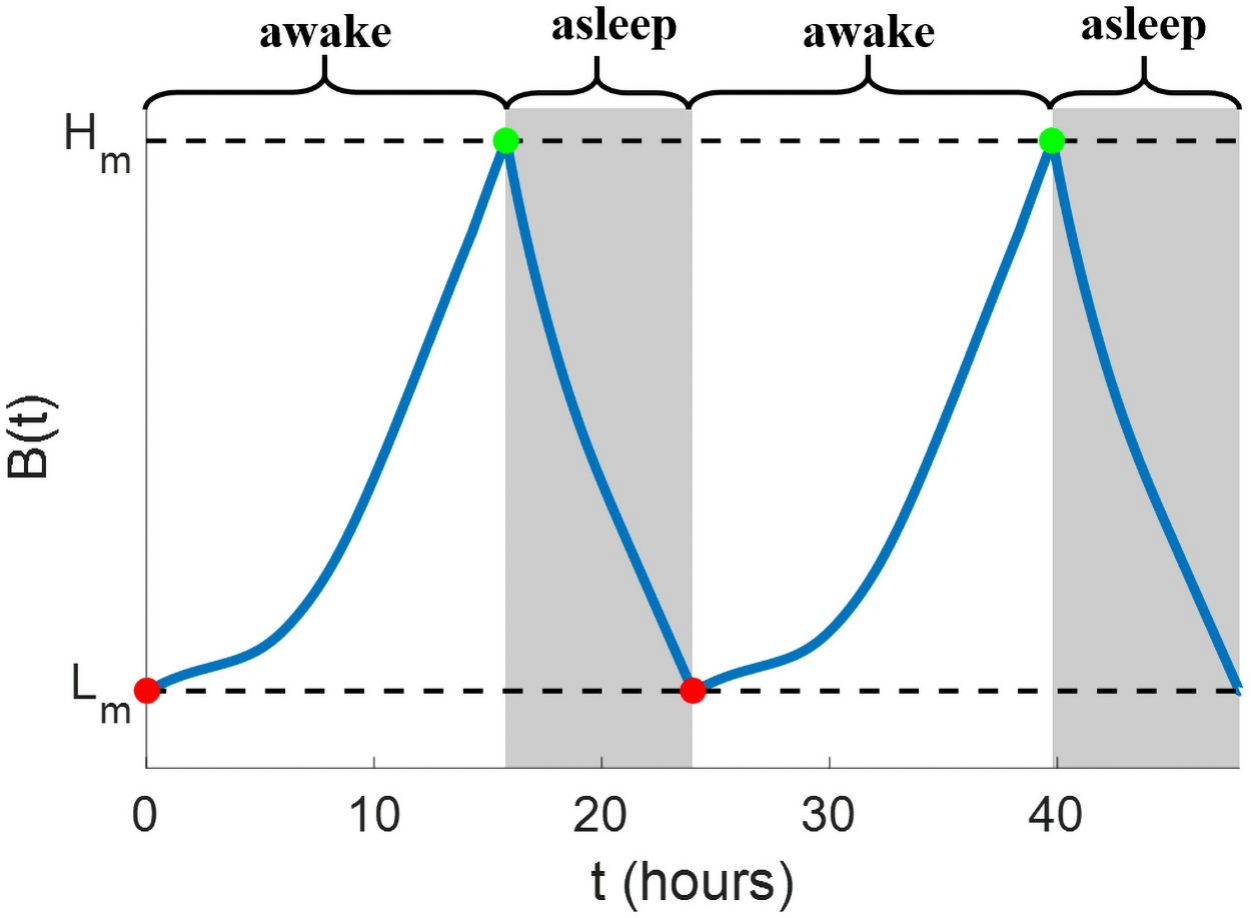
Illustrations of sleep state (gray region) and sleepiness (blue curve) under the spontaneous sleep schedule in Eq (9). The subject wakes up spontaneously when *B* = *L*_*m*_ (red nodes) and falls asleep spontaneously when *B* = *H*_*m*_ (green nodes).

The discrete dynamics of the sleep state based on sleepiness are demonstrated in Fig 2 and explicitly expressed as

$$\begin{array}{c} \beta \left(t\right)=F_{\beta }\left(y,\beta \left(t^{-}\right)\right)=\{\begin{array}{cc} 1, & t\in T_{\text{sleep}},\\ 0, & t\in T_{\text{wake}},\\ \beta (t^{-}), & \text{else}, \end{array} \end{array}$$ (9)

where *β*(*t*^−^) = lim_*τ*→*t*^−^_ *β*(*τ*), *t*^−^ represents the time just before *t* and *y* represents all continuous states in the two-process model. To incorporate the coupling impact of sleep state on the light and circadian rhythm, we replace the light-dependent term in Eq (1b) with

$$\begin{array}{c} \alpha \left(I\right)=\alpha_{0}\cdot {\left[\frac{I(1-\beta )}{I_{0}}\right]}^{p}. \end{array}$$ (10)

This equation implies that when *β*(*t*) = 1, we have *α*(*I*) = 0 and *u*(*t*) = 0. Thus, light has no impact on the Process L and the whole Process C when the subject is asleep.

In the following sections of this paper, the three two-process models are called *S+C3 model*, *S+C2 model*, and *S+C1 model*, respectively, with details listed in Table 2.

**Table 2:** Two-process models.

Model name	Model formulation	Description
S+C3	Eqs (1–2b), (6, 7), (10)	Full-order JFK model with Process S
S+C2	Eqs (2a, 2b), (3), (6, 7), (10)	2nd-order circadian rhythm model with Process S
S+C1	Eqs (3), (5, 6), (8), (10)	1st-order phase-reduced model with Process S

## 3 Entrainment strategies and problem formulation

When a periodic light input is used on the two-process model with the spontaneous sleep schedule, the two-process state converges to a stable trajectory with the same period. This phenomenon is called *entrainment*. Phase misalignment happens when the circadian and sleep rhythms of a subject deviate from the entrained rhythms corresponding to the ambient light-dark cycle, for example, in the case of jet-lag. In this paper, we look at three different re-entrainment strategies: open-loop entrainment with spontaneous sleep, minimum-time entrainment with spontaneous sleep, and minimum-time entrainment with controllable sleep.

### 3.1 Open-loop entrainment with spontaneous sleep schedule

As mentioned in Section 2.3, we denote the continuous state in the two-process model as *y*. For the S+C3 model, the state is expressed as *y* = [*n*, *x*, *x*_*c*_, *H*]^*T*^. Specifically, we consider a 24-hour periodic light input as:

$$\begin{array}{c} I_{\text{ref}}\left(t\right)=\{\begin{array}{cc} 1000\;\text{lux}, & \text{mod}(t,24)\in [0,16),\\ 0, & \text{mod}(t,24)\in [16,24), \end{array} \end{array}$$ (11)

which approximates the natural light-dark cycle on earth. The sunrise time *t* = 0 corresponds to 6 am and daily light is present between 6 am and 10 pm. Note that the natural light-dark duty cycle constantly changes depending on the latitude. We use the light pattern in Eq (11) as a simple approximation of the daily light pattern. The optimization methodology that we present in this paper does not depend on the particular reference light pattern. In terms of Eqs (1c) and (3), when *I* = 1000 lux, the corresponding steady value of *u* is 0.1731. For S+C2 and S+C1 models, the corresponding 24-hour periodic light input is represented as

$$\begin{array}{c} u_{\text{ref}}\left(t\right)=\{\begin{array}{cc} 0.1731, & \text{mod}(t,24)\in [0,16),\\ 0, & \text{mod}(t,24)\in [16,24). \end{array} \end{array}$$ (12)

The stable periodic solution of Eqs (1–2b), (6) with the spontaneous sleep schedule in Eq (9) and the periodic light input in Eq (11) is the entrained state for the S+C3 model, represented as *Y*_REF_(*t*) = [*n*_REF_(*t*), *x*_REF_(*t*), *x*_*c*REF_(*t*), *H*_REF_(*t*)]^*T*^. The entrained state *Y*_REF_ represents the circadian rhythm synchronized with the periodic light input in Eq (11). The corresponding periodic sleep state is represented as *β*_REF_(*t*). In this paper, the reference circadian rhythm that the entraining subject wants to reach is defined as

$$\begin{array}{c} y_{\text{ref}}\left(t\right)≜{\left[n_{\text{ref}}\left(t\right),x_{\text{ref}}\left(t\right),x_{c\text{ref}}\left(t\right),H_{\text{ref}}\left(t\right)\right]}^{T}=Y_{\text{REF}}\left(t+\Delta_{\text{init}}\right), \end{array}$$ (13)

where Δ_init_ represents the difference between the reference time at the beginning of entrainment (*t*_0_ = 0) and 6 am.

The existence of a periodic solution for the two-process model has been previously studied, e.g., in [35], where the authors presented a one dimensional function that maps the timing of sleep onset on one day to the timing of sleep onset on the following day. The fixed points of such function represent periodic solutions. In [23], the authors constructed a similar map for a more detailed sleep-wake regulation model that includes REM and NREM sleep stages.

One straightforward approach for entrainment is applying the spontaneous sleep schedule and reference light-dark cycle on the entraining subject directly, where the reference light-dark cycle is expressed as

$$\begin{array}{c} I\left(t\right)=I_{\text{ref}}\left(t+\Delta_{\text{init}}\right). \end{array}$$

As the light input is just a function of time, we call this entrainment the *open-loop entrainment*. The entrainment process is finished once some terminal conditions

$$\begin{array}{c} \varphi_{f}\left(y\left(t_{f}\right),t_{f}\right)\leq 0 \end{array}$$ (14)

are satisfied. The mathematical forms of *φ*_*f*_(*y*(*t*_*f*_), *t*_*f*_)≤0 for each model are given in Table 3, where the final tolerance tol is a small positive scalar, *t*_*f*_ is the final entrainment time, and ‖⋅‖_2_ represents the Euclidean norm. The state *n* is ignored in the terminal condition of the S+C3 model as it has a very fast time scale. The value of tol used in this paper will be discussed in S1 Appendix in details. The entrainment time of the open-loop entrainment is treated as a baseline for comparison.

**Table 3:** Entrainment formulation for two-process models.

Model	Continuous state *y*	State equation	Terminal condition *φ*_*f*_(*y*(*t*_*f*_), *t*_*f*_)≤0
S+C3	[*n*, *x*, *x*_*c*_, *H*]^*T*^	Eqs (1–2b), (6)	${\left\|{\left[x,x_{c},H\right]}^{T}-{\left[x_{\text{ref}},x_{c\text{ref}},H_{\text{ref}}\right]}^{T}\right\|}_{2}^{2}-\text{tol}\leq 0$
S+C2	[*x*, *x*_*c*_, *H*]^*T*^	Eqs (2a, 2b), (6)	${\left\|{\left[x,x_{c},H\right]}^{T}-{\left[x_{\text{ref}},x_{c\text{ref}},H_{\text{ref}}\right]}^{T}\right\|}_{2}^{2}-\text{tol}\leq 0$
S+C1	[*θ*, *H*]^*T*^	Eqs (5, 6)	${\left\|{\left[\theta ,H\right]}^{T}-{\left[\theta_{\text{ref}},H_{\text{ref}}\right]}^{T}\right\|}_{2}^{2}-\text{tol}\leq 0$

### 3.2 Minimum-time entrainment with spontaneous sleep schedule

In this case, the subject still falls asleep and wakes up spontaneously during the entrainment process. We want to find the optimal light input I*(*t*) ∈ [0, *I*_max_] ≜ Ω_*I*_ to drive *y*(*t*) to reach the terminal condition of entrainment in minimum time, where *I*_max_ is the maximum light intensity we can use during the entrainment process and Ω_*I*_ represents the feasible set of the light input. Given the initial condition *y*(0) = *y*_0_, the state equation

$$\begin{array}{c} \dot{y}=F\left(y,I,\beta \right), \end{array}$$ (15)

the sleep dynamics in Eq (9), and the reference state in (13), the minimum-time entrainment problem with spontaneous sleep is expressed in the following form:

$$\begin{array}{c} \text{minimize}\;t_{f},\\ \text{subject}\;\text{to}\;\text{the}\;\text{light}\;\text{constraint}\;I\left(t\right)\in \left[0,I_{\text{max}}\right],\\ \text{and}\;\text{the}\;\text{terminal}\;\text{condition}\;\varphi_{f}\left(y\left(t_{f}\right),t_{f}\right)=0, \end{array}$$

where *I*(*t*) is the optimization variable, the state *y*, state Eq (15), and terminal conditions of the three two-process models are listed explicitly in Table 3. We denote the minimizing *I*(*t*) as I*(*t*). (A detailed solution algorithm of the minimum-time entrainment problem with spontaneous sleep schedule is provided in the supplemental S1 Appendix).

### 3.3 Minimum-time entrainment with controllable sleep schedule

**Fig 3:**
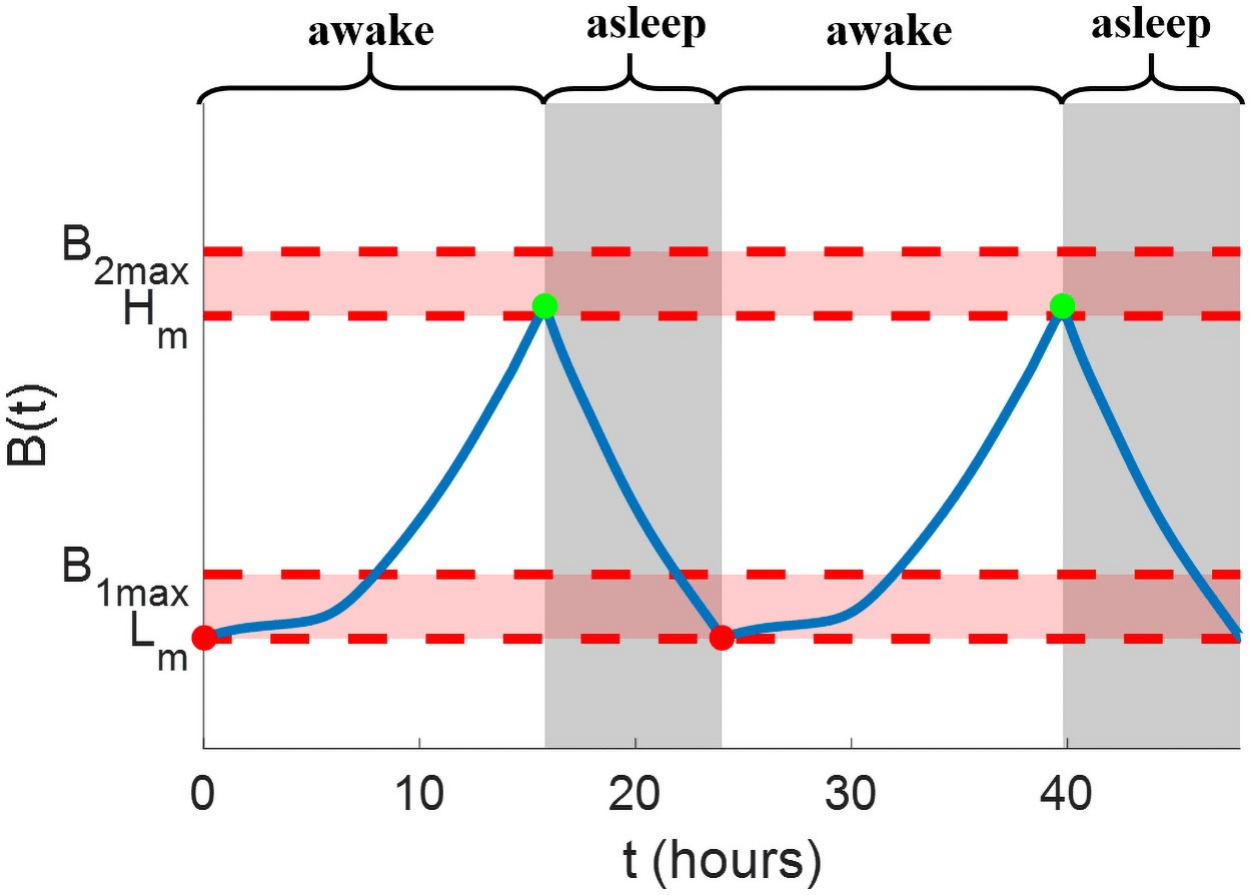
Illustrations of sleep state (gray region) and sleepiness (blue curve) under the controllable sleep schedule in (16) and (17). The two horizon pale red bars demonstrate the range of sleepiness that the subject could fall asleep and wake up.

Here we consider an alternative case. The subject could be woken up by an alarm clock or other means before waking up spontaneously, and when the subject begins to feel sleepy, he may remain awake. Thus, the value of *B*(*t*) at sleep and wake times could be higher than the thresholds *H*_*m*_ and *L*_*m*_. The dynamics of the sleep state are then given as

$$\begin{array}{c} \beta \left(t\right)=F_{\beta }^{\prime }\left(y,\beta \left(t^{-}\right)\right)=\{\begin{array}{cc} 1, & t\in \{T_{\text{sleep}}^{1},\ldots ,T_{\text{sleep}}^{N_{f}}\},\\ 0, & t\in \{T_{\text{wake}}^{1},\ldots ,T_{\text{wake}}^{M_{f}}\},\\ \beta (t^{-}), & \text{else}, \end{array} \end{array}$$ (16)

where $T_{\text{sleep}}^{i}$ and $T_{\text{wake}}^{j}$ represent the *i*-th sleep time and *j*-th wake time. The parameters *N*_*f*_ and *M*_*f*_ are total times of falling asleep and waking up during entrainment. Although the sleep schedule is controllable, we assume that it still should follow some constraints during entrainment: the entraining subject can only fall asleep when the value of sleepiness is high enough, i.e., $B(T_{\text{sleep}}^{i})\geq 0.67$, and cannot remain sleeping if the sleepiness is less than 0.17. The values of $B(T_{\text{sleep}}^{i})$ and $B(T_{\text{wake}}^{j})$ should also not be too high. Higher $B(T_{\text{sleep}}^{i})$ means the subject falls asleep later and higher $B(T_{\text{wake}}^{j})$ means the subject wakes up earlier, both of which could lead to sleep deprivation. Thus, the constraints for $B(T_{\text{sleep}}^{i})$ and $B(T_{\text{wake}}^{j})$ are given as

$$\begin{array}{c} 0.67\leq B\left(T_{\text{sleep}}^{i}\right)\leq B_{1max},\;0.17\leq B\left(T_{\text{wake}}^{j}\right)\leq B_{2max}, \end{array}$$ (17)

shown as Fig 3. In this case, we assume that the subject should remain awake for no longer than 18 hours every day. Hereafter, the upper bound for sleepiness at the sleep time is chosen as *B*_1*max*_ = 0.77, which is close to the value of sleepiness when the subject has stayed awake for about 2 hours after *B* = 0.67. The upper bound for sleepiness at the wake time is set as *B*_2*max*_ = 0.27, which corresponds to the sleepiness about 2 hours before *B* = 0.17. For this entrainment strategy, we want to find not only the light input but also the sleep schedule to entrain the circadian rhythm in minimum time. Given the initial condition *y*(0) = *y*_0_, the state equation in Eq (15), sleep dynamics in Eq (16), and reference state in Eq (13), the minimum-time entrainment problem with controllable sleep schedule is written as follows:

$$\begin{array}{c} \text{minimize}\;t_{f},\\ \text{subject}\;\text{to}\;\text{the}\;\text{light}\;\text{constraint}\;I\left(t\right)\in \left[0,I_{\text{max}}\right],\\ \text{the}\;\text{sleep}\;\text{time}\;\text{constraint}\;0.67\leq B\left(T_{\text{sleep}}^{i}\right)\leq 0.77,\forall i\in \left\{1,\ldots .,N_{f}\right\},\\ \text{the}\;\text{wake}\;\text{time}\;\text{constraint}\;0.17\leq B\left(T_{\text{wake}}^{j}\right)\leq 0.27,\forall j\in \left\{1,\ldots ,M_{f}\right\},\\ \text{and}\;\text{the}\;\text{terminal}\;\text{condition}\;\varphi_{f}\left(y\left(t_{f}\right),t_{f}\right)=0, \end{array}$$

where $I\left(t\right),T_{\text{sleep}}^{1},\ldots ,T_{\text{sleep}}^{N_{f}},T_{\text{wake}}^{1},\ldots ,T_{\text{wake}}^{M_{f}}$ are the optimization variables. We denote the corresponding optimizing solutions as $I^{*}\left(t\right),T_{\text{sleep}}^{1*},\ldots ,T_{\text{sleep}}^{N_{f}*},T_{\text{wake}}^{1*},\ldots ,T_{\text{wake}}^{M_{f}*}$. (A detailed solution algorithm of the minimum-time entrainment problem with controllable sleep schedule is provided in the supplemental S1 Appendix).

## 4 Application scenarios of circadian rhythm entrainment optimization

To demonstrate the application of our solution strategies (in S1 Appendix) in circadian rhythm entrainment optimization, we solve several entrainment cases of transmeridian travelers and shift workers in this section.

### 4.1 Entrainment of transmeridian travelers

In the case of transmeridian travelers, the reference state that travelers want to reach is given as *y*_ref_(*t*) = *Y*_REF_(*t* + Δ_init_) with Δ_init_ = 1, which means the entrainment process begins at 7 am at the destination. The initial state of travelers is set as *y*(0) = *y*_ref_(Δ_shift_), where Δ_shift_ ∈ [0, 24] represents the time shift between home and the destination (home in advance/westward travel).

#### 4.1.1 Minimum-time entrainment with spontaneous and controllable sleep schedule

**Fig 4:**
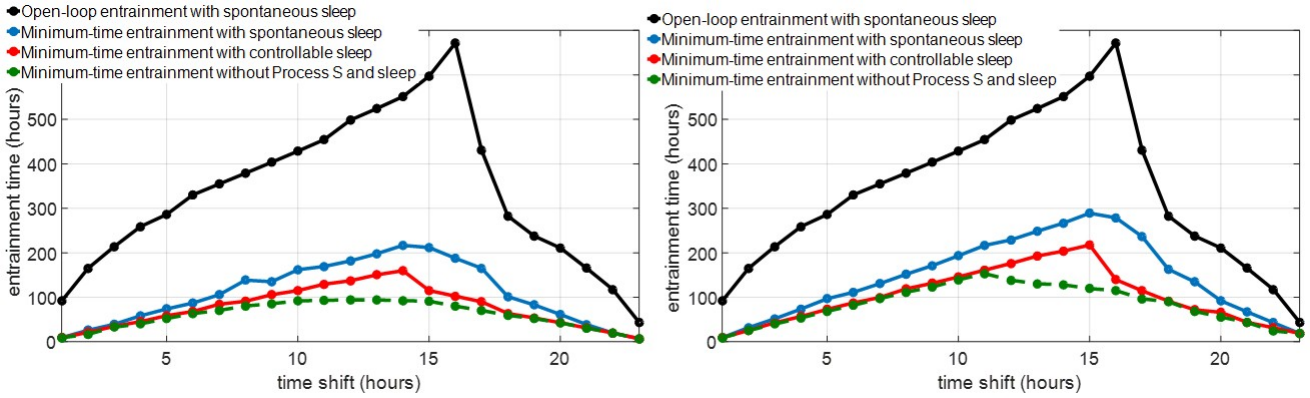
Entrainment time comparison of different entrainment strategies with *I*_max_ = 10000 lux (left), *I*_max_ = 1000 lux (right) and different time shifts Δ_shift_. The black curve shows the open-loop entrainment time with spontaneous sleep; the blue curve shows the minimum entrainment time with spontaneous sleep; the red curve shows the minimum entrainment time with controllable sleep; the green dash line shows the minimum entrainment time of circadian rhythm without Process S and sleep, solved by the method in [15].

In this section, we evaluate the cases with Δ_shift_ ∈ {1, 2, …, 23} for the S+C3 model. The three entrainment strategies mentioned in Sections 3.1, 3.2 and 3.3 are simulated and the solution algorithms in S1 Appendix are performed to calculate the optimal light for minimum-time entrainment with spontaneous sleep and optimal light and sleep for minimum-time entrainment with controllable sleep. Fig 4 shows the entrainment time by these three entrainment strategies for different time shifts and maximum light intensities. Compared with the open-loop entrainment strategy (black curves), the entrainment time is decreased significantly by optimizing the light input without changing the spontaneous sleep schedule (blue curves), e.g., in *I*_max_ = 10000 lux entrainment cases, the entrainment time is shortened by 30–450 hours. The entrainment time is further decreased by optimizing both light and sleep schedule (red curves), especially in cases with 8–18 hours time shift where the entrainment time is further decreased by about 50 hours. For cases with *I*_max_ = 1000 lux, the entrainment process is also significantly shortened by optimizing both light and sleep schedule, e.g., the entrainment time is reduced from 279 hours (in the minimum-time entrainment with spontaneous sleep) to 140 hours in the case of 16 hours shift.

**Fig 5:**
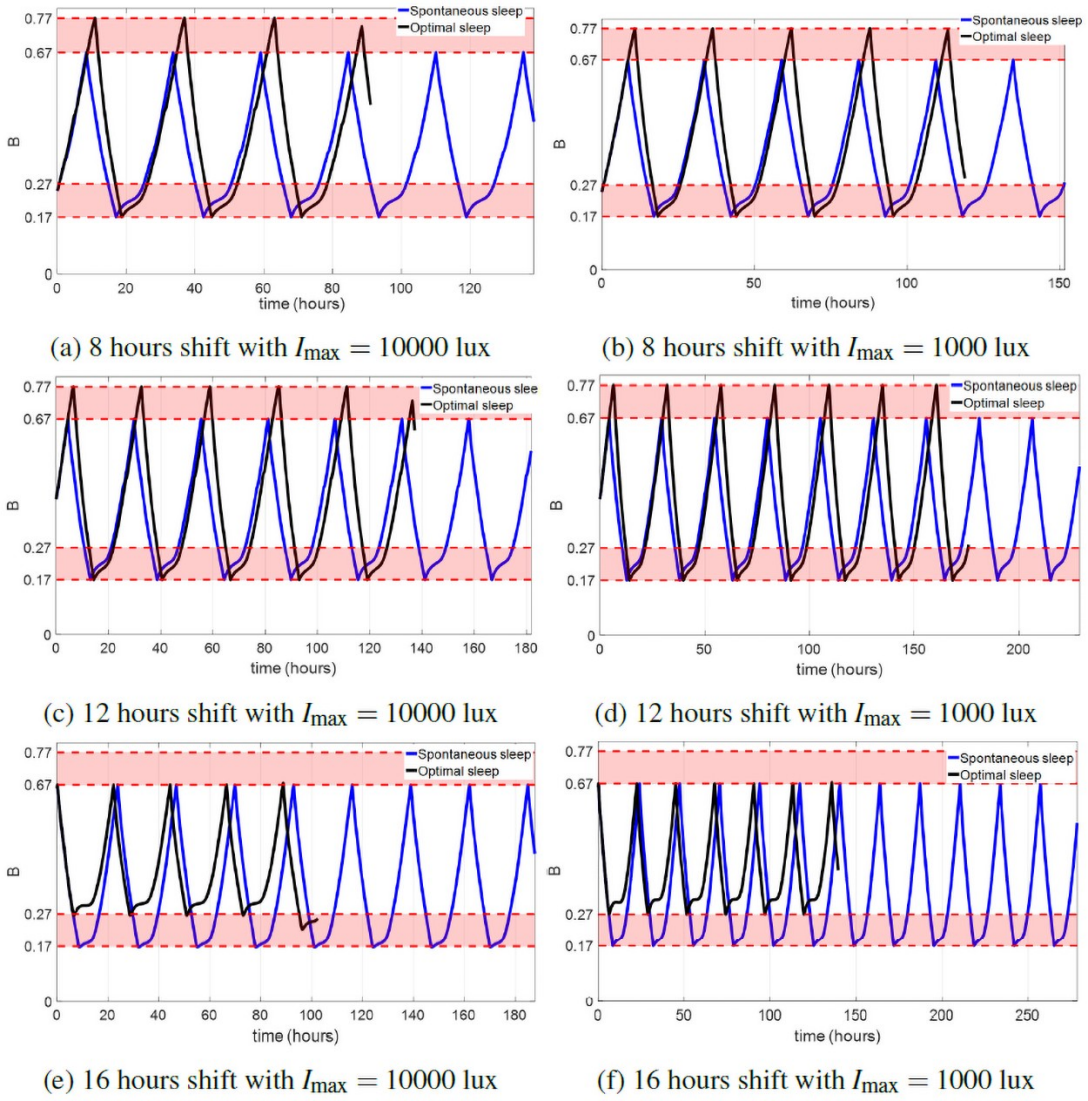
Sleepiness comparison of the minimum-time entrainment with spontaneous sleep (blue curves) and with optimal (controllable) sleep (black curves). In this figure, we only plot the sleepiness value during the entrainment process (i.e., before reaching the terminal condition in Eq (14)). The black curves terminate earlier than the blue curves, emphasizing that the entrainment times with optimal sleep are shorter than those with spontaneous sleep.

**Fig 6:**
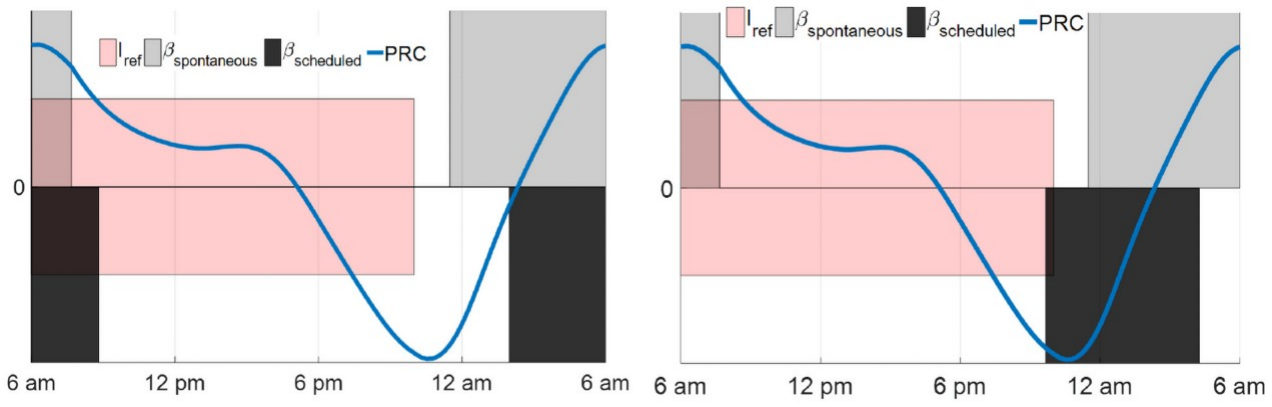
**(Left) Scheduled sleep with *B*_sleep_ = 0.77, *B*_wake_ = 0.17**. (Right) Scheduled sleep with *B*_sleep_ = 0.67, *B*_wake_ = 0.27. The red region represents that the reference light is present between 6 am and 10 pm, the gray region in each figure shows the spontaneous sleep period, the blue line shows the value of phase response curve. The black region in the left panel shows the scheduled sleep period that the subject wakes up when *B* = 0.17 and falls asleep when *B* = 0.77; the black region in the right panel shows the sleep period that the subject wakes up when *B* = 0.27 and falls asleep when *B* = 0.67.

Fig 5 compares the sleepiness of some minimum-time entrainment cases with spontaneous sleep and optimal (controllable) sleep schedules. The maximum time costs in the minimum-time entrainment with controllable sleep with *I*_max_ = 10000 lux and *I*_max_ = 1000 lux both occur at about 14–15 hours shift. In 8 and 12 hours shift cases with the optimal sleep schedule, the entraining subject wakes up spontaneously (*B* = 0.17) but remains awake until *B* = 0.77; while in 16 hours shift cases with either *I*_max_ = 10000 lux and *I*_max_ = 1000 lux, the subject falls asleep spontaneously but wakes up earlier (when *B* = 0.27) than normal. These results imply that during the minimum-time entrainment process, the circadian rhythm and sleep schedule are delayed by delaying the sleep times but keeping the spontaneous wake times, while advanced by advancing the wake times but keeping the spontaneous sleep times. These phenomena are explained by the phase response curve and reference sleep state shown in Fig 6: In the periodic spontaneous sleep-wake cycle, the daily wake period starts from 7:30 am to 11:10 pm. The phase response value is positive in the time region between 7:30 am and 5 pm, and negative between 5 pm and 11:10 pm. The subject tends to fall asleep in the phase-delay region and wake up in the phase-advance part. Fig 6 also shows the scheduled sleep periods by delaying the sleep time (left) and advancing the wake time (right), respectively. Delaying the sleep time increases the phase-delay region and decreases the phase-advance region in the wake period while advancing the wake time results in a larger phase-advance region in the wake period compared with the spontaneous wake period. Therefore, using optimized sleep/wake times with optimal light input accelerates the entrainment processes and takes shorter entrainment time than only using optimal light input with the spontaneous sleep schedule.

#### 4.1.2 Comparison with minimum-time entrainment results without Process S and sleep-wake cycle

**Fig 7:**
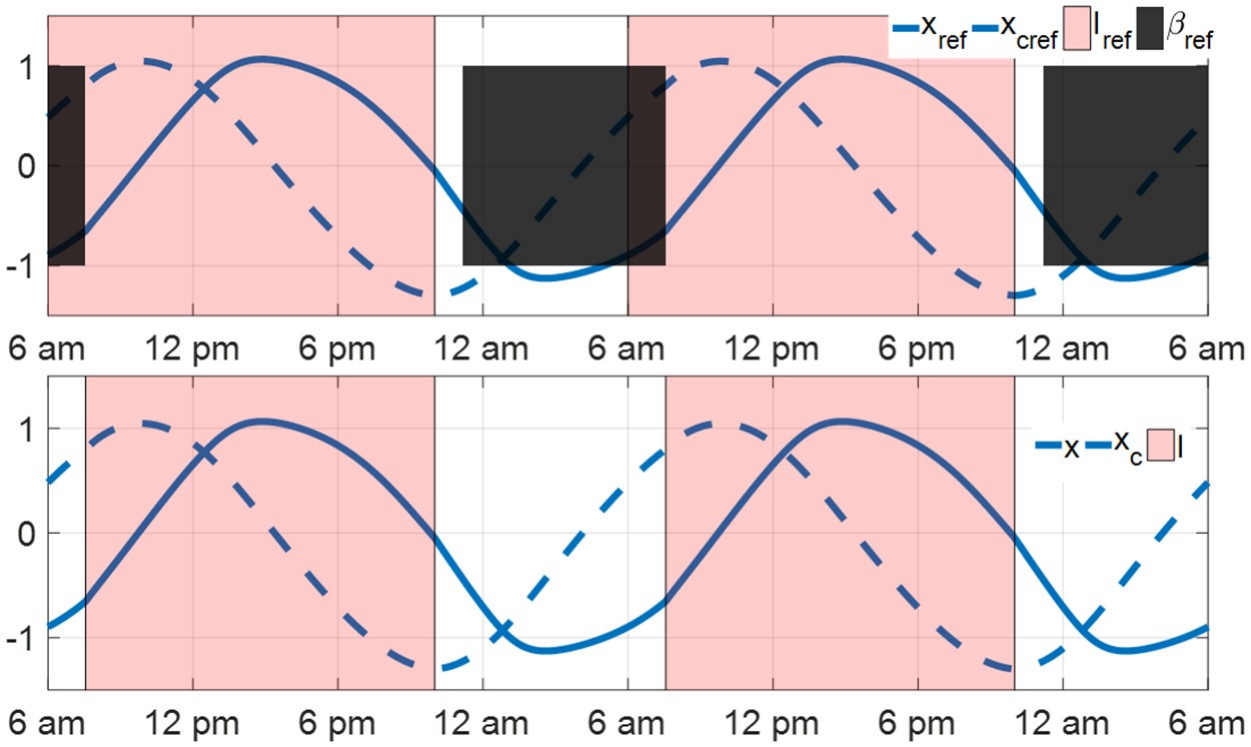
The periodic circadian state of the JFK model with (upper) and without (lower) Process S and sleep-wake cycle. The red region in the upper panel shows the reference daily light is present between 6 am and 10 pm, the black region in the upper panel indicates the reference sleep period is from about 11:10 pm to 7:30 am. The red region in the lower panel shows the actual daily light that the reference subject receives is from 7:30 am to 10 pm.

The periodic circadian state [*x*_ref_(*t*), *x*_*c*ref_(*t*)] of the S+C3 model is plotted in the upper panel in Fig 7. During the interval between 6 am and 7:30 am, the subject is sleeping but the light is present. Therefore, the actual light-dark cycle that the reference subject receives is 14.5–9.5-hour. The periodic solution of the full-order JFK model under the 14.5–9.5-hour light-dark cycle without Process S and sleep-wake cycle is plotted in the lower panel in Fig 7, which is equal to the reference value [*x*_ref_(*t*), *x*_*c*ref_(*t*)]. The minimum-time entrainment problem on the JFK model (Process C) without Process S and sleep has been discussed in [12, 15]. We treat the periodic solution in the lower panel in Fig 7 as the reference trajectory and calculate the optimal light input for the minimum-time entrainment of JFK circadian rhythm model by the method in [15]. The minimum entrainment time is plotted as the green dashed lines in Fig 4, where the maximum entrainment time in minimum-time entrainment cases occurs at around 12-hour shift. However, the maximum entrainment time of the S+C3 model in minimum-time entrainment cases occurs around 14–16 hours shift, shown on the blue and red curves in Fig 4. These results imply that after taking Process S and the sleep-wake cycle into account, the minimum-time circadian rhythm entrainment becomes asymmetrical, in which recovery from traveling eastward tends to be more time-consuming than from traveling westward.

Denote the optimal light in the case of only entraining Process C as $I_{C}^{*}\left(t\right)$. We apply $I_{C}^{*}\left(t\right)$ on the S+C3 model by $I\left(t\right)=I_{C}^{*}\left(t\right)$. To guarantee that the subject receives all light input during the entrainment process, the sleep schedule is tuned to fit the optimal light schedule as follows:

$$\begin{array}{c} \beta \left(t\right)=\{\begin{array}{cc} 0, & I_{C}^{*}\left(t\right)>0\;\text{or}\;B\left(t\right)\leq 0.17,\\ 1, & I_{C}^{*}\left(t\right)=0\;\text{and}\;B\left(t\right)\geq 0.67,\\ \beta (t^{-}), & \text{else}. \end{array} \end{array}$$ (18)

**Fig 8:**
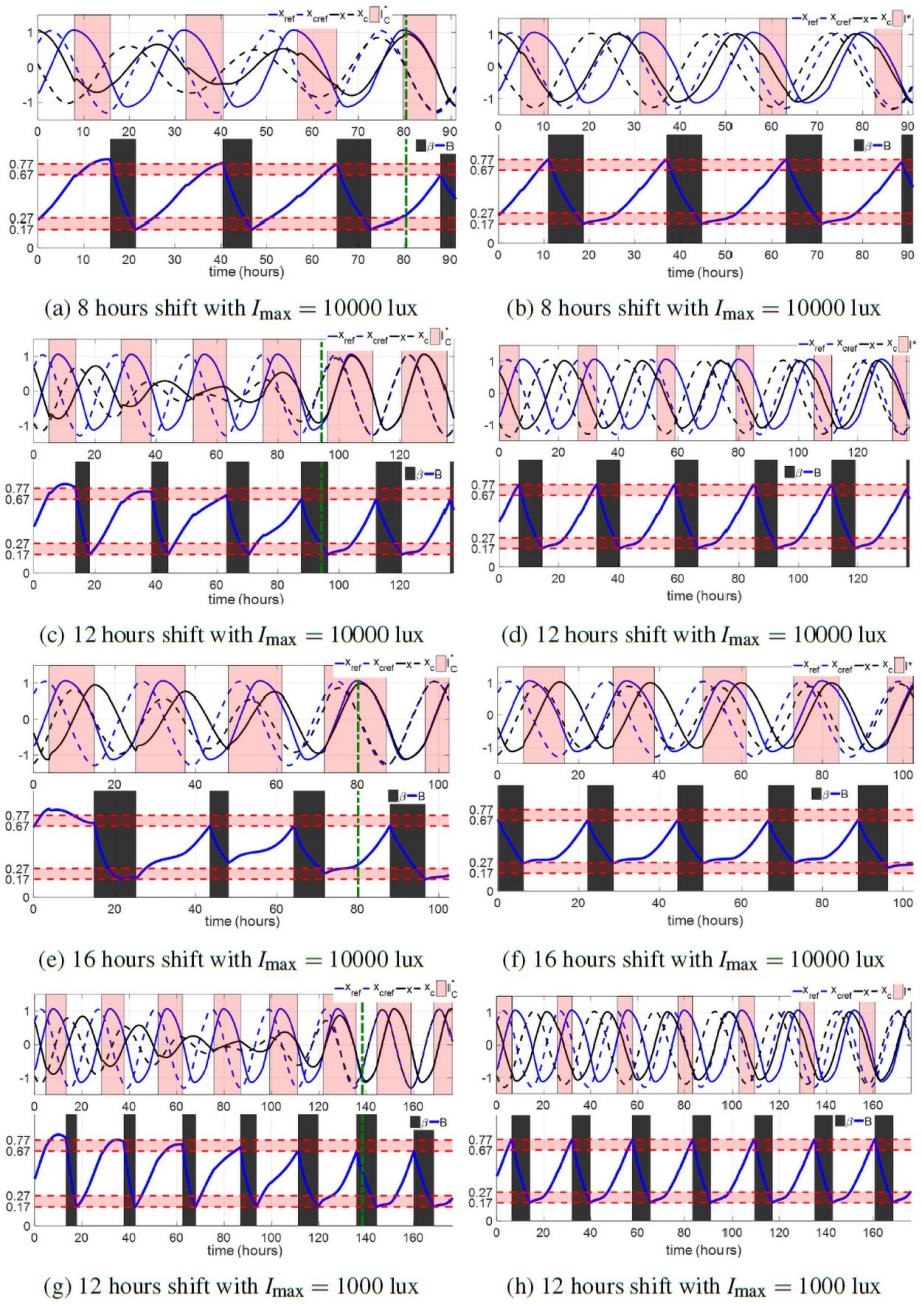
Minimum-time entrainment cases with the tuned sleep schedule in Eq (18) (left column) and controllable sleep schedule (right column). The upper panel of every sub-figure shows the reference states (blue curves), entraining states (black curves) as well as the light input. Note that the optimal lights from the gradient descent process in this paper are all bang-off controls, i.e., I*(*t*) = 0 or *I*_max_. In the following figures, we represent the light-on region in the form of a vertical pale red bar. The lower panel of every sub-figure demonstrates the sleep period (black region) and sleepiness (blue curves) during entrainment. The green dashed lines in left subfigures indicate the entrainment time.

The left subfigures (a), (c), (e) and (g) in Fig 8 show four entrainment cases with $I\left(t\right)=I_{C}^{*}\left(t\right)$ and the tuned sleep schedule in (18) for 8, 12, 16 hours shift with *I*_max_ = 10000 lux and 12 hours shift with *I*_max_ = 1000 lux. The amplitudes of the core body temperature oscillators are all quenched in these cases. This phenomenon is called the *minimum path shifting* in [12], which is more likely to happen in entrainment using bright light. The maximum value of sleepiness during the wake periods reaches up to 0.81 in these cases, and the wake periods are longer than 20 hours in some days during entrainment. For several days, the subject only sleeps for 4–5 hours. Short sleep durations, especially those less than 6 hours, have been associated with several health problems [36]. Even though the total entrainment time could be shorter, the optimal light of the JFK circadian rhythm model without consideration of Process S and the sleep-wake cycle is impractical for the entraining subject. The sleep constraints in (17) should be taken into account in entrainment solution strategies. We perform the solution algorithm in S1 Appendix for the S+C3 model and solve the optimal light and sleep schedule for the minimum-time entrainment of these cases, shown in right subfigures (b), (d), (f) and (h) in Fig 8. The entrainment time of the four cases in Fig 8 increases from 80.44, 94.22, 80.06, and 138.40 hours to 91.03, 137.20, 102.50, and 176.00 hours, respectively, i.e., the entrainment time of these four cases increases by about half to two days with Process S and sleep/wake time constraints. However, daily sleep intervals are guaranteed to be about 6–8 hours, the optimal sleep schedule also avoids excessive sleepiness (no larger than 0.77) during entrainment. Minimum-time entrainment results with controllable sleep also show that the amplitude of the circadian oscillator is no longer quenched in the minimum-time entrainment even under a bright light of 10000 lux. Under this schedule, the core body temperature runs in the periodic reference limit cycle without amplitude quenching.

#### 4.1.3 Model simplification

**Fig 9:**
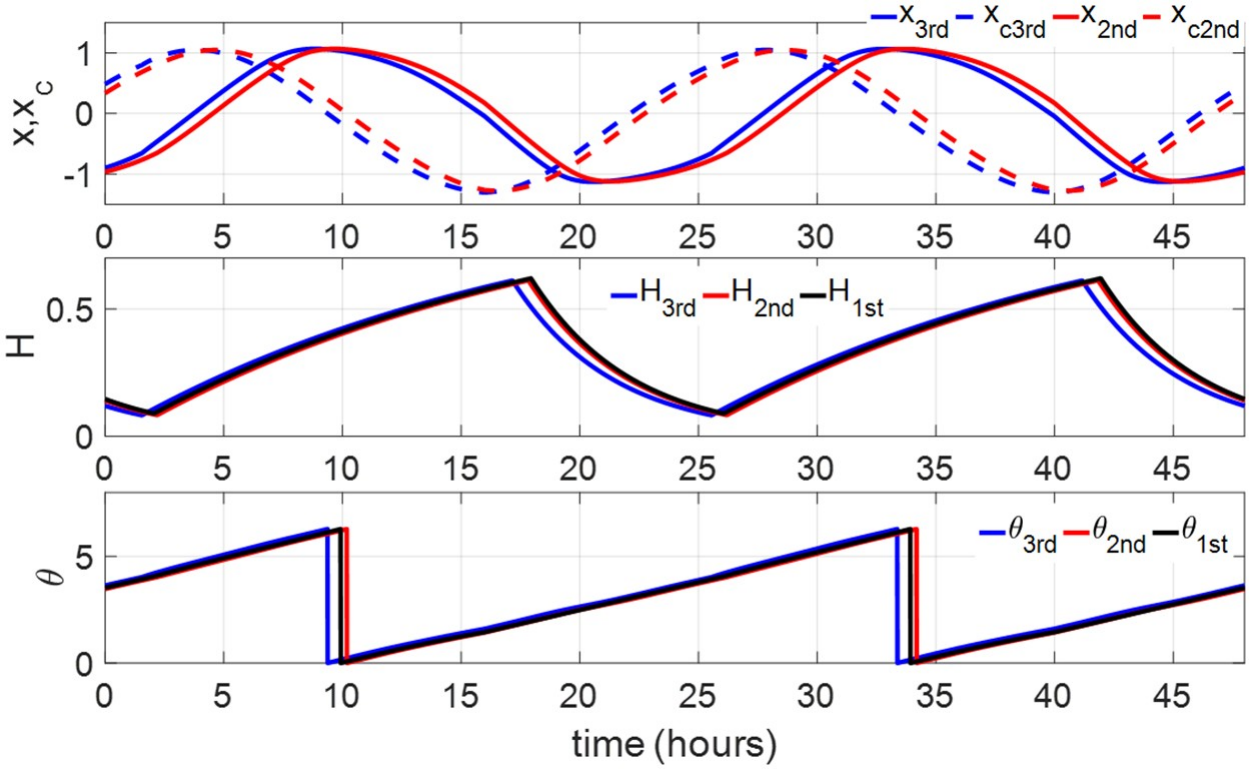
Periodic circadian and sleep homeostasis states in three models. The blue lines of *x*_3rd_, *x*_*c*3rd_, *H*_3rd_, *θ*_3rd_ represent the periodic circadian and sleep homeostasis states of the S+C3 model, the red lines of *x*_2nd_, *x*_*c*2nd_, *H*_2nd_, *θ*_2nd_ represent those of the S+C2 model, the black lines of *θ*_1st_ and *H*_1st_ represent the periodic circadian phase and sleep homeostasis of the S+C1 model.

The light inputs of S+C2 model and S+C1 model are given by *u* ∈ [0, *u*_max_], where *u*_max_ = 0.2208 and 0.1731 correspond to *I*_max_ = 10000 and 1000 lux based on Eq (3). The periodic solutions of all three two-process models under the 16–8-hour light-dark cycle in (11) or (12) are plotted in Fig 9, which shows that these periodic solutions are close to each other.

The minimum-time entrainment problem of the two simplified two-process models are studied, and the optimal light input and sleep schedule of these two models are represented as *u*_2nd_(*t*), *β*_2nd_(*t*), *u*_1st_(*t*), *β*_1st_(*t*), respectively. We adapt and apply these optimal solutions to the S+C3 model as follows

$$\begin{array}{c} I\left(t\right)=\frac{I_{\text{max}}\times u_{1\text{st}}\left(t\right)}{u_{\text{max}}},\;\beta \left(t\right)=\{\begin{array}{cc} 0, & \beta_{1\text{st}}\left(t\right)=0\;\text{and}\;B\left(t\right)\leq 0.27,\\ 1, & \beta_{1\text{st}}\left(t\right)=1\;\text{and}\;B\left(t\right)\geq 0.67,\\ 0, & B(t)\leq 0.17,\\ 1, & B(t)\geq 0.77,\\ \beta (t^{-}), & \text{else}. \end{array} \end{array}$$ (19a)

$$\begin{array}{c} I\left(t\right)=\frac{I_{\text{max}}\times u_{2\text{nd}}\left(t\right)}{u_{\text{max}}},\;\beta \left(t\right)=\{\begin{array}{cc} 0, & \beta_{2\text{nd}}\left(t\right)=0\;\text{and}\;B\left(t\right)\leq 0.27,\\ 1, & \beta_{2\text{nd}}\left(t\right)=1\;\text{and}\;B\left(t\right)\geq 0.67,\\ 0, & B(t)\leq 0.17,\\ 1, & B(t)\geq 0.77,\\ \beta (t^{-}), & \text{else}. \end{array} \end{array}$$ (19b)

**Fig 10:**
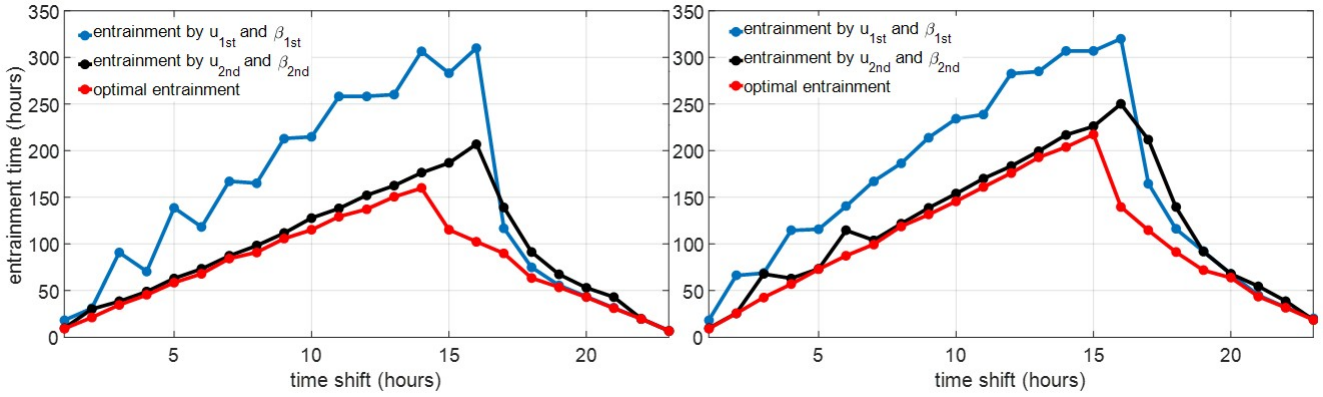
Entrainment time by applying the optimal light and sleep schedule of the simplified models on the full model with *I*_max_ = 10000 lux (left) and *I*_max_ = 1000 lux (right).

As *u*_1st_(*t*) and *u*_2nd_(*t*) are all bang-off controls, i.e., *u*_1st_(*t*) ∈ {0, *u*_max_} and *u*_2nd_(*t*) ∈ {0, *u*_max_}, the lights in Eqs (19a) or (19b) are also bang-off with *I*(*t*) ∈ {0, *I*_max_}. If *y*(*t*) does not converge to *y*_ref_(*t*) by strategies in (19a) and (19b), we use *I*_ref_(*t* + Δ_init_) and the spontaneous sleep as the supplement strategy after the final time of *u*_1st_(*t*) and *u*_2nd_(*t*). Fig 10 shows the entrainment time when applying the optimal light and sleep schedule of the S+C2 model or S+C1 model on the S+C3 model, in which the red lines represent the minimum entrainment time of the S+C3 model with the optimal light and sleep schedule, the blue and black lines show the entrainment time by the light and sleep schedule in (19a) and (19b). The entrainment time of (19b) is closer to the optimal entrainment time of the S+C3 model than the entrainment time of (19a) is, in most cases. It is obvious that compared with the 2nd-order circadian rhythm model, which only ignores Process L, the 1st-order circadian phase model in Section 2.1.3 is reduced further and results in a larger difference at the final optimal solution and entrainment time. The large difference between the entrainment time using *u*_1st_/*β*_1st_ or *u*_2nd_/*β*_2nd_ and the optimal entrainment time in some cases is also attributed to the long entrainment time using the reference light and spontaneous sleep schedule after the final time of *u*_1st_/*β*_1st_ and *u*_2nd_/*β*_2nd_. Further, the optimal light and sleep schedule are used on the S+C3 model in an open-loop form. To fix the error brought by model simplification, we introduce the feedback entrainment controller in the next section.

#### 4.1.4 Feedback implementation for minimum-time entrainment

Even though the optimal solution from the S+C1 model does not work well on the full S+C3 model in an open-loop form, we still observe that during the minimum-time entrainment process with the optimal sleep schedule, the amplitude of the circadian state is not quenched too much even under a bright light of 10,000 lux, as shown in Fig 8. This suggests the possibility of representing the oscillatory variables (*x*, *x*_*c*_) as only a phase variable *θ*, and designing the optimal light and sleep schedule only by using the circadian phase and *H*(*t*) [37]. The circadian phase is defined based on *x* and *x*_*c*_ as in Eq (4). The periodic reference state [*x*_ref_(*t*), *x*_*c*ref_(*t*), *n*_ref_(*t*), *H*_ref_(*t*)]^*T*^ is also simplified as a reference phase, defined as

$$\begin{array}{c} \theta_{\text{ref}}\overset{\Delta }{=}\{\begin{array}{cc} \text{arctan}(\frac{-x_{c\text{ref}}}{x_{\text{ref}}}), & \text{if}\;x_{\text{ref}}\geq 0,\\ \text{arctan}(\frac{-x_{c\text{ref}}}{x_{\text{ref}}})+\pi , & \text{if}\;x_{\text{ref}}<0, \end{array}\theta_{\text{ref}}\in \left[0,2\pi \right). \end{array}$$ (20)

The feedback entrainment controller is defined in the form of (21), where the functions Φ_wake_ and Φ_sleep_ represent the feedback entrainment strategy during wake and sleep periods. The inputs of the feedback controller are entraining and reference phase, *H*(*t*) and current sleep state. The state *n*(*t*) is ignored in the feedback implementation as Process L is a transient process and has very small effects on the entrainment strategy. The function Φ_wake_ outputs light *I*(*t*) and sleep schedule Γ_sleep_, which represents how much time the subject has before he should fall asleep; the function Φ_sleep_ only outputs a wake-up schedule Γ_wake_, i.e., how many hours the subject has before he should wake up.

$$\begin{array}{c} \left[I\left(t\right),\Gamma_{\text{sleep}}\left(t\right)\right]=\Phi_{\text{wake}}\left(\theta \left(t\right),H\left(t\right),\theta_{\text{ref}}\left(t\right)\right)\;\text{when}\;\beta \left(t\right)=0,\\ {}[\Gamma_{\text{wake}}\left(t\right)]=\Phi_{\text{sleep}}\left(\theta \left(t\right),H\left(t\right),\theta_{\text{ref}}\left(t\right)\right)\;\text{when}\;\beta \left(t\right)=1. \end{array}$$ (21)

The procedure of formulating this feedback entrainment controller is listed as follows:

**Step 1**: For each value of *I*_max_, define Δ_init_ = 1, Δ_shift_ = {1, 2, …, 23}. Calculate the optimal light and sleep for minimum-time entrainment, and generate 23 sets of optimal trajectory $\left[\theta^{*}\left(t\right),H^{*}\left(t\right),\theta_{\text{ref}}^{*}\left(t\right),I^{*}\left(t\right),\Gamma_{\text{sleep}}^{*}\left(t\right)/\Gamma_{\text{wake}}^{*}\left(t\right)\right]$;

**Step 2**: Sample these optimal trajectories with a sampling time Δ*t* = 0.01h and generate training data points. The training dataset is separated into two parts: the wake dataset [*θ*, *H*, *θ*_ref_, *I*, Γ_sleep_]_wake_ contains all data points with *β* = 0 and the sleep dataset [*θ*, *H*, *θ*_ref_, Γ_wake_]_sleep_ corresponds to all data points with *β* = 1;

**Step 3**: Perform a nearest neighbor algorithm to formulate the functions Φ_wake_ and Φ_sleep_ by [*θ*, *H*, *θ*_ref_, *I*, Γ_sleep_]_wake_ and [*θ*, *H*, *θ*_ref_, Γ_wake_]_sleep_, respectively.

To evaluate the performance of the feedback entrainment controller calculated by the process above, we simulate some new entrainment cases as the cross-validation set:

**Case a**: A traveler flies from one place to another with a time shift Δ_shift_ valued in {1h, 2h, …, 23h}. The initial entrainment time *t*_0_ = 0 corresponds to 10 am local time at home, i.e., *y*(0) = *Y*_REF_(4). The initial state of the traveler is given as *x*(0) = 0.0815, *x*_*c*_(0) = 1.0459, *H*(0) = 0.1984, *n*(0) = 0.6631 and *y*_ref_(*t*) = *Y*_REF_(*t* + Δ_init_) with Δ_init_ = 4 − Δ_shift_;

**Case b**: A traveler flies from one place to another with a time shift Δ_shift_ valued in {1h, 2h, …, 23h}. The initial entrainment time *t*_0_ = 0 corresponds to 10 pm local time at home, i.e., *y*(0) = *Y*_REF_(16). The initial state of the traveler is given as *x*(0) = −0.0479, *x*_*c*_(0) = −1.3003, *H*(0) = 0.5854, *n*(0) = 0.6838 and *y*_ref_(*t*) = *Y*_REF_(*t* + Δ_init_) with Δ_init_ = 16 − Δ_shift_.

**Fig 11:**
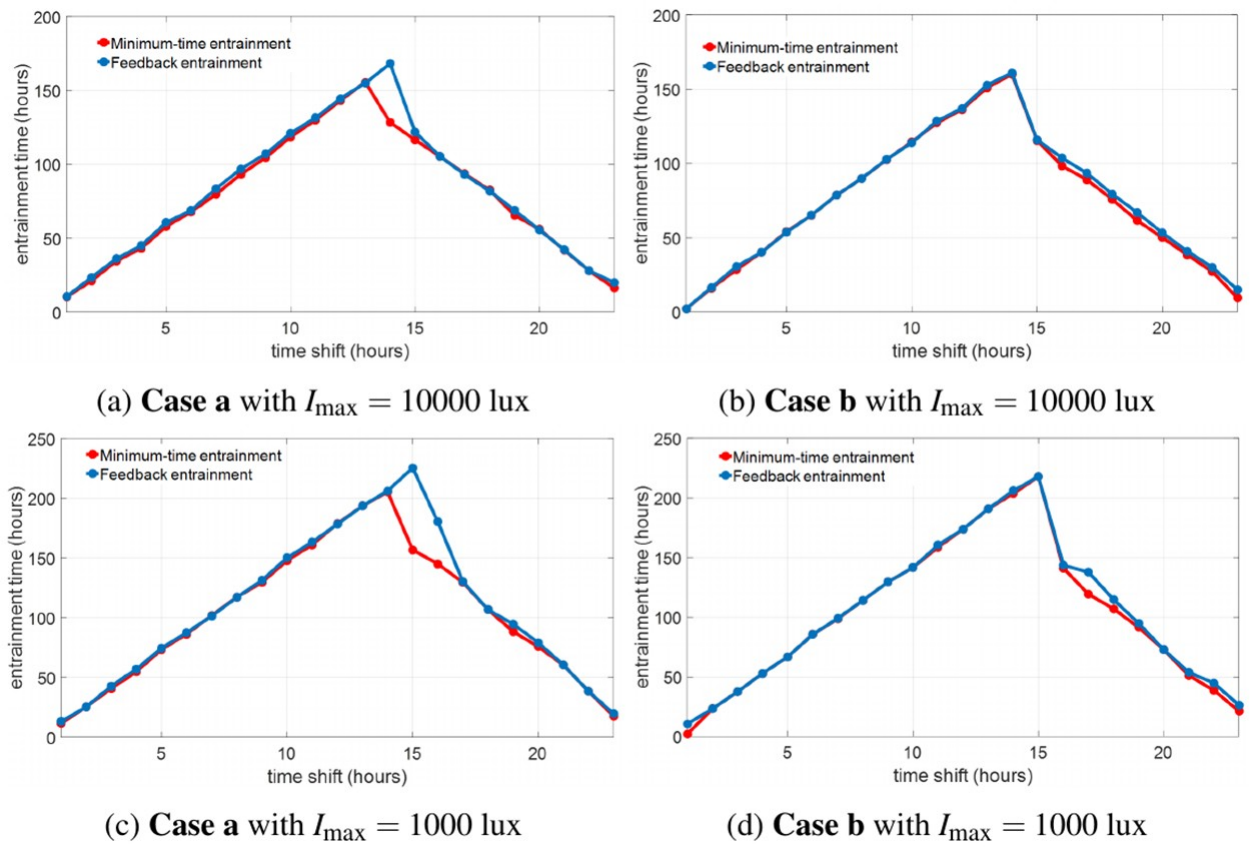
Comparison of the minimum-time (red) and feedback entrainment (blue) time on the cross-validation entrainment cases. We can observe that, in most cases, the feedback entrainment time is close or equal to the minimum entrainment time.

The training cases in **Step 1** fix the initial reference states *y*(0) = *Y*_REF_(1) but change the initial entraining state *y*(0) = *Y*_REF_(1 + Δ_shift_) by choosing different Δ_shift_, i.e., the training cases simulate the entrainment processes that the travelers fly from different time zones to the same destination. The cross-validation set **Case a** and **Case b**, with fixed initial states of the traveler (*y*(0) = *Y*_REF_(4) in **Case a** and *y*(0) = *Y*_REF_(16) in **Case b**) for different time shifts, simulate the entrainment processes that the traveler flies from home to different destinations. The feedback entrainment time plotted as the blue curves in Fig 11 is very close to the optimal (minimum) entrainment time (red curves) in most cases. This indicates the feedback light input and sleep schedule in (21) are similar to the optimal light and sleep in the minimum-time entrainment of these cases. The feedback entrainment controller generated from the procedure we propose above functions robustly on entrainment cases with various initial conditions.

### 4.2 Entrainment of shift workers

Here we consider the entrainment problem of shift workers based on the S+C3 model. The experiments in [28, 29] asked the shift workers to stay awake between 8 pm and 8 am in one night under a constant artificial light with intensity *I*_shift_. In Section 4.2.1, the entrainment processes of shift workers in [28] are simulated under three values of *I*_shift_: 0 lux, 100 lux (working under a dim light) and 1000 lux (working under a bright light with the same intensity as the reference daylight). Note that 0 lux light exposure can be achieved, practically, by wearing a pair of circadian-light blocking goggles to filter all light with wavelengths less than 480 nm that could suppress the melatonin secretion. The open-loop entrainment and minimum-time entrainment with the controllable sleep schedule (after night shifts) of shift workers are studied and discussed. In Section 4.2.2, we assume the light input during the night shift is also controllable. The optimal light input during and after the night shift and the sleep schedule after the night shift are calculated for minimum-time entrainment of the shift workers.

#### 4.2.1 Entrainment of shift workers with constant light during night shift

**Fig 12:**
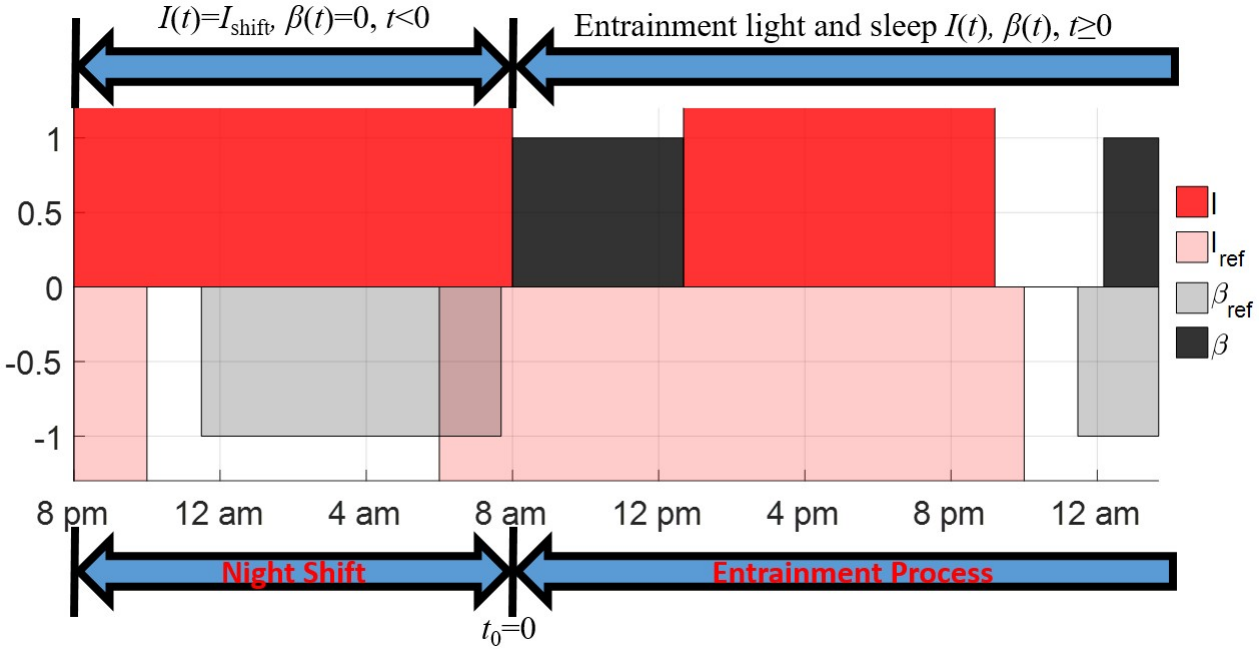
Illustrations of the entrainment process of shift workers under constant light during the night shift from 8 pm to 8 am. The entrainment process starts at the end of the night shift, i.e., *t*_0_ = 0 corresponds to 8 am. The red region represents the time when the light is on for the shift worker, the light red region represents the time when the reference daylight is present, the black region shows the sleep period of shift workers and the gray region shows the sleep period of the reference subject.

In this entrainment case, we set *t* = −12 to correspond to the beginning of the night shift (8 pm), the initial time of entrainment *t* = *t*_0_ = 0 corresponds to the end of the night shift (8 am), as demonstrated in Fig 12. The reference state we want the shift worker to reach is given as *y*_ref_(*t*) = *Y*_REF_(*t* + 2). Note that the expression *t* + 2 here corresponds to the fact that the entrainment begins at 8 am. The circadian state at the beginning of night shift is given as *y*(−12) = *y*_ref_(−12) and the light input and sleep state during the night shift are expressed as

$$\begin{array}{c} I\left(t\right)=I_{\text{shift}},\;\beta \left(t\right)=0,\;\forall t\in \left[-12,0\right). \end{array}$$

**Fig 13:**
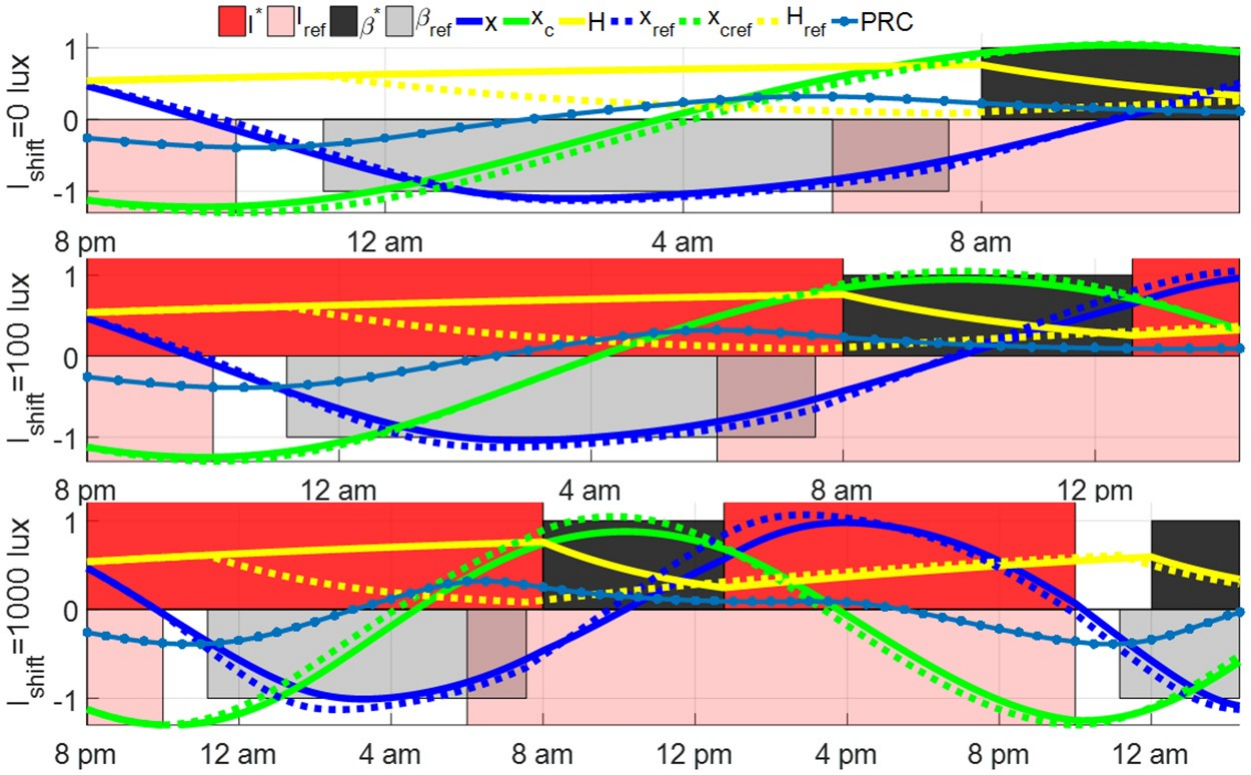
Open-loop entrainment with different night shift light intensity *I*_shift_. The solid curves show the time courses of the entraining state of night shift workers, and the dashed curves show the time courses of the reference state. The open-loop entrainment time is 3.46 hours for *I*_shift_ = 0 lux, 6.29 hours for *I*_shift_ = 100 lux and 18.32 hours for *I*_shift_ = 1000 lux. The blue dot line shows the phase response curve (PRC) of the entraining state.

The open-loop entrainment strategy in Section 3.1 is applied to the shift worker by taking the reference light *I*(*t*) = *I*_ref_(*t* + 2) and spontaneous sleep schedule directly. The open-loop entrainment processes with *I*_shift_ = 0, 100, 1000 lux are plotted in Fig 13. Working with goggles during the night shift results in the shortest entrainment time among these three night shift lighting conditions. Experimental results in [28, 29] demonstrated that compared with the night shift in complete darkness or wearing the circadian-light blocking goggles, the night shift in bright light with 1000 lux suppressed the melatonin production and disturbed circadian rhythm more. The simulation results in this part indicate that using the goggles during the night shift shortens the entrainment time following the night shift. Note that even if the shift worker’s Process C maintains synchrony with that of the reference at the end of the shift, his Process S will lose synchronization due to sleep deprivation. A recent work by Piltz et al [38, 39] showed that recovery from sleep deprivation under a spontaneous schedule can take a long time.

**Fig 14:**
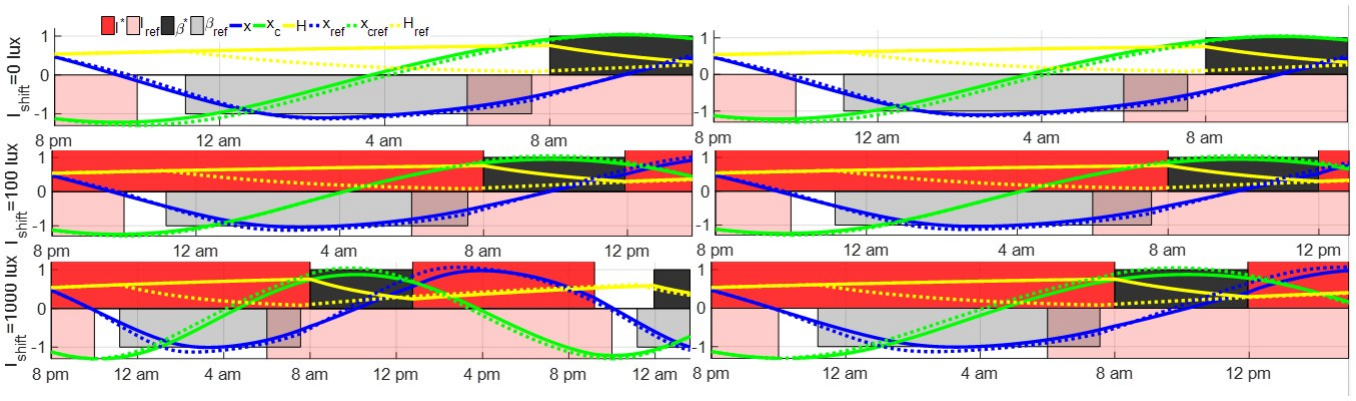
Minimum-time optimal entrainment with controllable sleep schedule and different night shift light intensity *I*_shift_, with maximum entrainment light intensity *I*_max_ = 1000 lux (left) or *I*_max_ = 10000 lux (right). The minimum entrainment time of these cases is given as 3.46 hours for *I*_shift_ = 0 lux and *I*_max_ = 1000 lux, 5.80 hours for *I*_shift_ = 100 lux and *I*_max_ = 1000 lux, 17.62 hours for *I*_shift_ = 1000 lux and *I*_max_ = 1000 lux, 3.46 hours for *I*_shift_ = 0 lux and *I*_max_ = 10000 lux, 4.80 hours for *I*_shift_ = 100 lux and *I*_max_ = 10000 lux and 6.96 hours for *I*_shift_ = 1000 lux and *I*_max_ = 10000 lux, respectively.

The light input and sleep schedule after the night shift are optimized and the results of minimum-time entrainment with controllable sleep and various *I*_shift_ and *I*_max_ are plotted in Fig 14. These results demonstrate that wearing the goggles during the night shift still results in shorter entrainment time compared with the other two lighting conditions. Compared with open-loop entrainment results in Fig 13, the entrainment time is decreased by optimizing the light input and sleep schedule after night shifts in several cases, e.g., in the case with *I*_shift_ = 1000 lux and *I*_max_ = 10000 lux, the entrainment time is decreased from 18.32 hours to 6.96 hours. In the entrainment case with *I*_shift_ = 0 lux, as the entrainment is finished during the first sleep period following the night shift, the light and sleep schedule cannot be further optimized.

**Fig 15:**
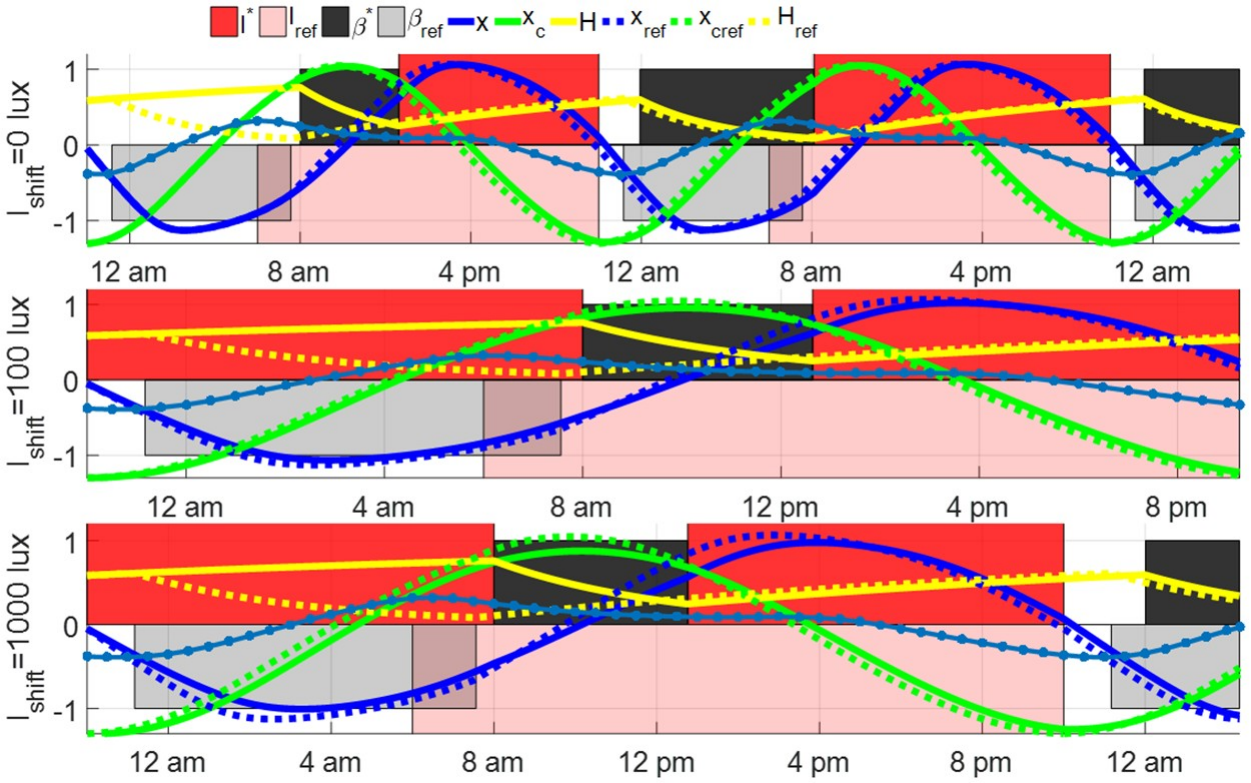
Open-loop entrainment with different lighting condition *I*_shift_ during night shift between 10 pm to 8 am, where the open-loop entrainment time is 44.06 hours for *I*_shift_ = 0 lux, 13.25 hours for *I*_shift_ = 100 lux and 18.32 hours for *I*_shift_ = 1000 lux.

**Fig 16:**
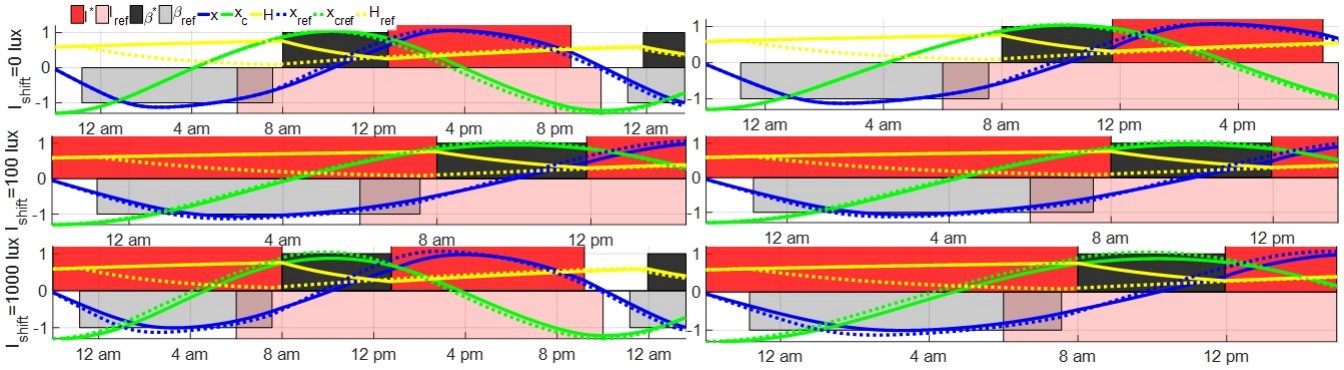
Minimum-time optimal entrainment of shift workers with night shift from 10 pm to 8 am with controllable sleep schedule and different working light intensity *I*_shift_, maximum light intensity *I*_max_ = 1000 lux (left) and *I*_max_ = 10000 lux (right). The minimum entrainment time of these cases is given as 17.68 hours for *I*_shift_ = 0 lux and *I*_max_ = 1000 lux, 6.47 hours for *I*_shift_ = 100 lux and *I*_max_ = 1000 lux, 17.62 hours for *I*_shift_ = 1000 lux and *I*_max_ = 1000 lux, 11.38 hours for *I*_shift_ = 0 lux and *I*_max_ = 10000 lux, 5.60 hours for *I*_shift_ = 100 lux and *I*_max_ = 10000 lux and 6.96 hours for *I*_shift_ = 1000 lux and *I*_max_ = 10000 lux, respectively.

We also study the impact of the shift schedule on the results. We vary the schedule by assuming that the night shift is between 10 pm and 8 am. The initial condition of this night shift is *y*(−10) = *y*_ref_(−10). The open-loop entrainment and minimum-time entrainment results are plotted in Figs 15 and 16. Different from results in Figs 13 and 14, the dim lighting condition with *I*_shift_ = 100 lux results in the fastest entrainment among the three light conditions when the night shift starts at 10 pm, in both open-loop entrainment cases and minimum-time entrainment cases. The difference in the entrainment results between the 8 pm-8 am night shift cases and the 10 pm-8 am ones can be explained by the phase response curve, plotted as blue dot lines in Figs 13 and 15. The phase response curve in Fig 13 shows the reference subject gets the reference light from 8 pm-10 pm (phase-delay region with negative PRC value) and 8 am-12:30 pm (phase-advance region with positive PRC value) while the entraining shift worker keeps away from light input during these two intervals. The phase-advancing impact of the reference light from 8 am-12:30 pm (approximately the first wake time of shift workers) is slightly larger than the phase-delay impact of the reference light from 8 pm-10 pm. If the constant light intensity during the night shift from 8 pm to 8 am is *I*_shift_ = 100 or 1000 lux, the light between 8 pm-8 am delays the phase of circadian state, drives the entraining state away from the reference state and, as a result, prolongs the entrainment process following the night shift. Thus the case with *I*_shift_ = 0 lux is less time-consuming compared with the other two cases. While in the case with a night shift between 10 pm and 8 am in Fig 15, the reference light between 8 am and 12:30 pm on the first day after the night shift advances the reference circadian phase while the shift worker is sleeping during this time. To compensate for the loss of the phase-advancing light in this interval, the dim light *I*_shift_ = 100 lux during 10 pm and 8 am is used to shorten the entrainment process. Increasing or decreasing *I*_shift_ leads to growth in the entrainment time.

#### 4.2.2 Minimum-time entrainment of shift workers with optimal light during night shift

**Fig 17:**
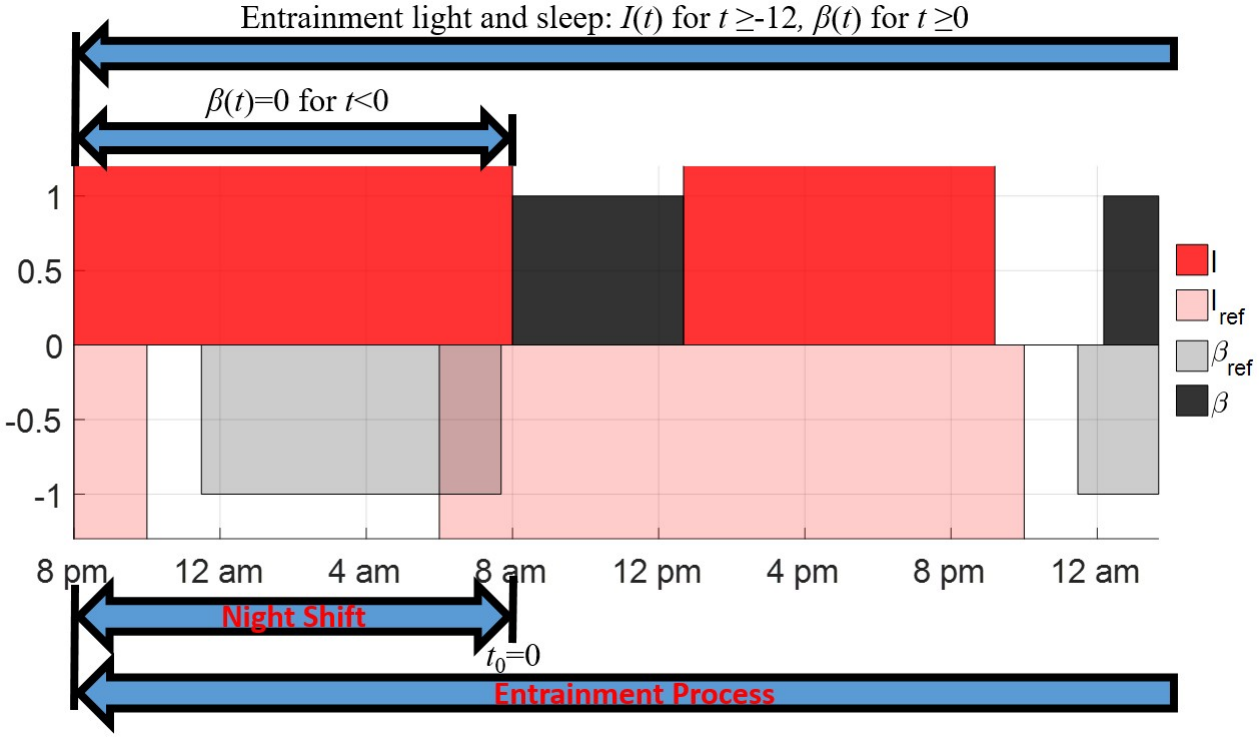
Illustrations of the entrainment process of the shift worker with controllable light during the night shift. The entrainment process starts at the beginning of the night shift.

As discussed in Section 4.2.1, the light during the night shift has significant impacts on the entrainment time. If the light during the night shift is also controllable, as illustrated in Fig 17, we optimize the light input both during and after the night shift as well as the sleep schedule after the night shift to minimize the entrainment time.

**Fig 18:**
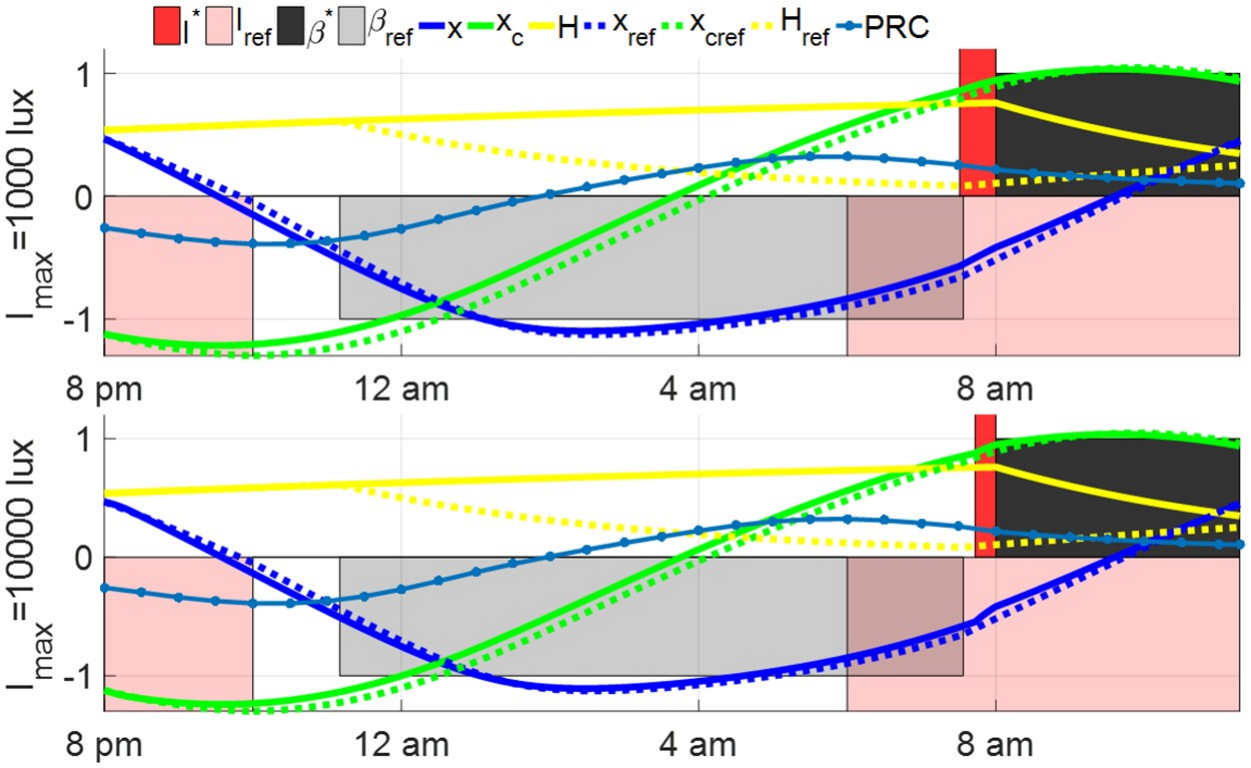
Minimum-time entrainment with optimal light input during the night shift from 8 pm to 8 am with *I*_max_ = 1000 lux (upper) and *I*_max_ = 10000 lux (lower). The entrainment time of these two cases is both 3.28 hours (12 hours night shift is excluded from the entrainment time).

**Fig 19:**
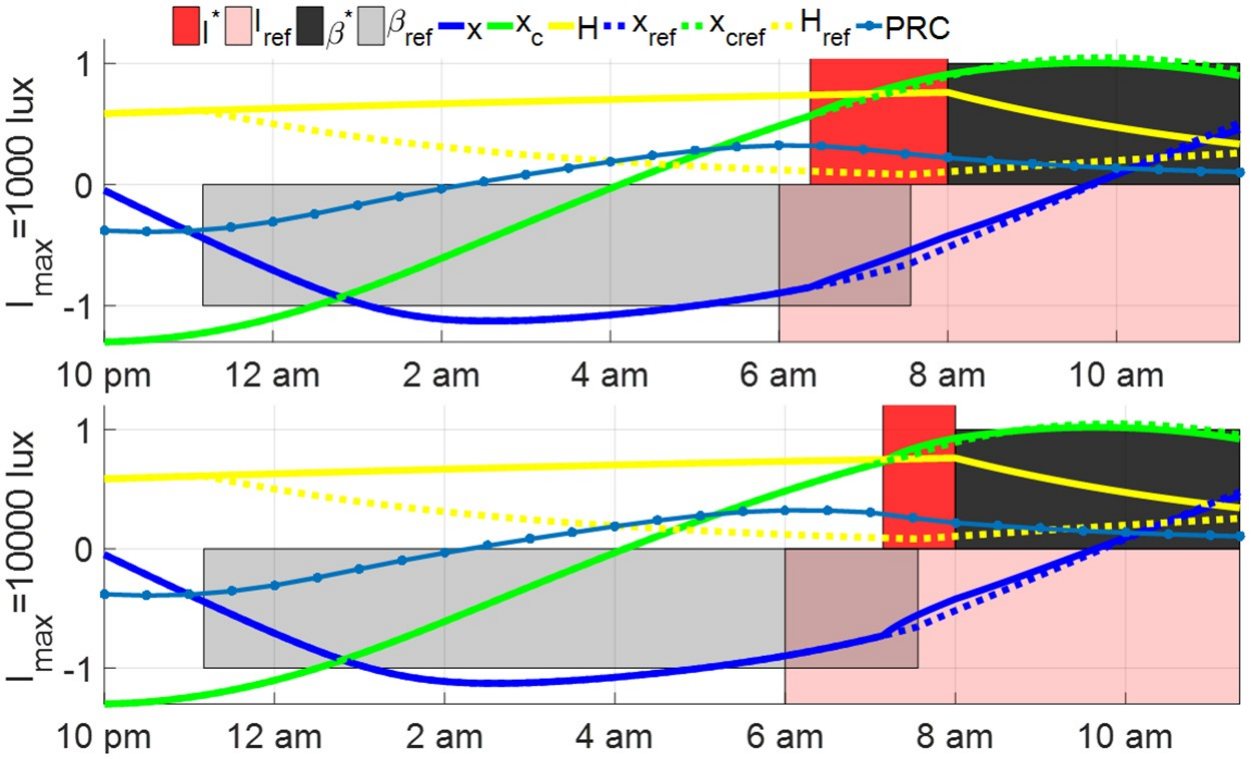
Minimum-time entrainment with optimal light input during the night shift from 10 pm to 8 am with *I*_max_ = 1000 lux (upper) and *I*_max_ = 10000 lux (lower). The entrainment time of these two cases is both 3.46 hours (10 hours night shift is excluded from the entrainment time).

The optimal light input during the night shift from 8 pm to 8 am for minimum-time entrainment is given in Fig 18. In the cases with maximum light intensity *I*_max_ = 1000 lux and *I*_max_ = 10000 lux, the entrainment time is shortened to 3.28 hours (entrainment ends at around 11:17 am). In the minimum-time entrainment case with *I*_max_ = 1000 lux, the shift worker remains under 1000 lux light without goggles for about 30 minutes towards the end of the night shift, while in the case with *I*_max_ = 10000 lux, the shift worker remains under 10000 lux light without goggles for about 17 minutes. As mentioned in Section 4.2.1, the reference light from 8 pm to 10 pm and 8 am to 11:17 am slightly advances the reference circadian phase. Therefore, to compensate for the small phase-advance impact of the reference light on reference subject, the shift worker should get light input in a very short interval with positive PRC value during the minimum-time entrainment, as shown in Fig 18. The minimum-time entrainment results with optimal light during the night shift from 10 pm to 8 am are shown in Fig 19, in which the shift worker receives 1000 lux light for about 98 minutes or 10000 lux light for about 51 minutes towards the end of the night shift. This scheme results in an entrainment time of 3.46 hours (entrainment ends at around 11:28 am). This night shift worker only keeps away from the reference light during the interval between 8 am and 11:28 am, which is a phase-advancing interval according to the PRC. Thus, he should stay under light for a longer time with positive PRC value during minimum-time entrainment compared with the shift worker in Fig 18.

## 5 Discussion and conclusions

This paper solves the minimum-time entrainment problem on the two-process model that combines the circadian rhythm model (the JFK model) with the sleep process model. Using the calculus of variations and a gradient descent method, we determine the optimal light input and sleep schedule for minimum-time entrainment of transmeridian travelers and shift workers under different maximum entrainment light intensities, time shifts, and night shifts. Different from previous works, we take Process S and sleep-wake cycle into circadian rhythm entrainment and treat the controllable sleep schedule as an optimization variable.

The simulations of the entrainment cases of transmeridian travelers in this paper demonstrate that: (a) Compared with the open-loop entrainment by natural (reference) light, the optimal lighting strategy significantly decreases the entrainment time. The entrainment time is further shortened by optimizing both the light input and the sleep schedule; (b) The daily (optimized) sleep period is about 6–8 hours in the minimum-time entrainment results with the controllable sleep schedule. In contrast, the optimal entrainment results in [12, 15] ignore Process S and the sleep-wake cycle, resulting in sleep periods of only 4–5 hours in several cases, which could result in excessive sleepiness and make the entrainment process impractical; (c) Applying the optimal light and sleep schedule from the simplified two-process models on the full two-process model directly is ineffective, resulting in longer entrainment times. However, we also propose a feedback controller that is learned from the optimal entrainment results of these simplified models, and demonstrate that it performs well and it is robust against the error resulting from model simplification in various entrainment cases.

The simulations of the entrainment cases of night shift workers in this paper demonstrate that: (a) The open-loop entrainment time of night shift workers wearing circadian-light blocking goggles during 8 pm and 8 am is shorter than that of workers in bright light. This result is related to the conclusion in [28] that wearing these goggles during night shifts helps maintain the circadian rhythm; (b) The entrainment time of shift workers may be shortened by optimizing the light input and sleep schedule after the night shift; (c)The lighting condition during the night shift also has significant impacts on the entrainment time. Appropriately setting the light intensity (or wearing goggles) during the night shift also shortens the entrainment time of shift workers.

The amplitude suppression in the core body temperature oscillator is closely connected with the minimum-time entrainment with bright light (with intensity higher than 1000 lux) in previous literature [12, 15, 40]. However, the phenomenon of CBT amplitude suppression may also be attributed to some health-related issues: the experimental results in [41] demonstrated the CBT oscillator tends to flatten with increasing age. The work in [42] showed that the quenched CBT amplitude is related to sleep disruption. Simulation results in Section 4.1.2 and Fig 8 in this paper demonstrate that the optimal light solution without consideration of sleep could lead to quenched CBT amplitude, excessive sleepiness (*B*(*t*) ≥ 0.8), and short sleep duration (shorter than 5 hours), while the optimal light and sleep schedule from the two-process model avoid quenched amplitude and ensure the daily sleep duration is not shorter than 6 hours and maximum sleepiness (*B*(*t*)) is not larger than 0.77. These simulation results also indicate the quenched amplitude has a close connection with the sleep disruption during entrainment.

Real-time application of the results reported in this paper requires the ability to compute the optimal light and sleep schedules in real-time. To this end, we have developed a feedback-based implementation of the optimal schedules, which does not require executing the functional gradient descent optimization in real-time. The feasibility of the feedback-based implementation is supported by two assumptions. First, we have the two-process model that accurately represents the subject. Second, the states of the two-process models can be measured or observed. Regarding the first assumption, we note that both the JFK circadian rhythm model and Achermann’s two-process model used in this paper were formulated based on previous experimental data from human subjects. However, the sleeping habit and biological features vary between subjects and therefore mathematical models with fixed parameters may not match individual circadian rhythms. As for the second assumption, the states of the two-precess model are typically not directly observable. However, we envision designing state observers [43] that can provide state estimations based on some observables. These observables can be provided by wearable sensors, such as Actiwatch and portable core body temperature monitor [44], which have been developed to measure the circadian and sleep features. Further, they can also facilitate the efforts to personalize the models. The measurement data from these sensors may be used in model calibration and design of personal light and sleep schedule for entrainment.

## Supporting information

S1 Appendix Terminal condition for entrainment process and solution strategies. (PDF)
