# Supplementary material for: Optimization of light exposure and sleep schedule for circadian rhythm entrainment
Source: PLoS One. 2021 Jun 8;16(6):e0251478. doi: 10.1371/journal.pone.0251478 (PMC8186815; doi:10.1371/journal.pone.0251478)
Supplement: S1 Appendix — (PDF) [file pone.0251478.s001.pdf]

Terminal condition for entrainment process and solution strategies

Eq (6) implies that the state $y(t)$ in the two-process model follows different dynamics when the subject is sleeping ($\beta = 1$) and awake ($\beta = 0$), i.e., the two-process model is piecewise smooth and contains finite modes (i.e., sleeping and awake intervals). In this paper, we apply variational calculus to determine the piecewise extremal solution for entrainment with either spontaneous sleep or controllable sleep. Assume the dynamics of the piecewise smooth model in the i-th mode is given in the following form:

$$\dot{y} = F_i(y, I),\ t \in [t_{i-1}, t_i),\ \forall i \in \{1, 2, \cdots, N\}, \tag{0.1}$$

where N is the total number of modes during the entrainment process. The initial condition is defined at the beginning of the 1st mode as $t_0 = 0,\ y(0) = y_0$. The objective function is given in an integral function spans the whole entrainment process as:

$$J(I; y_0) = \int_{t_0}^{t_f} L(\tau, y, I) d\tau, \tag{0.2}$$

where the integrand L maps the point $(t, y(t), I(t))$ to a real number.

0.1 Terminal condition for entrainment process

Since the S+C3 model is a deterministic dynamical system with a stable limit cycle under the reference light in Eq (11), the solution trajectory under the reference light should converge to the reference trajectory. For the entrainment problems of the S+C3 model, the terminal condition at the final time t_f of entrainment processes is given as

$$\varphi_f(y(t_f), t_f) = \|[x, x_c, H]^T(t_f) - [x_{\text{ref}}, x_{c\text{ref}}, H_{\text{ref}}]^T(t_f)\|_2^2 - \text{tol} \leq 0. \tag{0.3}$$

In this paper, the final tolerance is set to tol $= 0.01$, which corresponds to a 20-minute difference in the circadian phase shift. Note that the final sleep state $\beta(t_f)$ could be different from the reference sleep state $\beta_{\text{ref}}(t_f) = \beta_{\text{REF}}(t_f + \Delta_{\text{init}})$. We assume that when the entraining state is close to the reference state, as shown in Eq (0.3), the entraining subjects are able to adjust their sleep schedule to the reference sleep schedule rapidly by themselves. We use randomized numerical simulations and probability theory to justify (0.3) with tol=0.01 as the terminal condition of the entrainment process for the S+C3 model: assume the light input and sleep schedule of the entraining subject after he reaches the terminal condition are reference light and spontaneous sleep, we uniformly randomly choose 10000 sets of initial conditions $[x(0), x_c(0), H(0)]$ on the spheres satisfying

$$\|[x, x_c, H]^T(0) - [x_{\text{ref}}, x_{c\text{ref}}, H_{\text{ref}}]^T(0)\|_2^2 = 0.01,$$
$$[x_{\text{ref}}, x_{c\text{ref}}, H_{\text{ref}}](t) = [x_{\text{REF}}, x_{c\text{REF}}, H_{\text{REF}}](t + \Delta),\ \Delta \in [0, 24],$$

where Δ is uniformly sampled between 0 and 24 and $n(0)$ is uniformly valued between 0 and 1 for every case, then simulate the S+C3 model forward. The state error is defined as

$$e(t) = \|[x, x_c, H]^T(t) - [x_{\text{ref}}, x_{\text{cref}}, H_{\text{ref}}]^T(t)\|_2^2.$$

The left subfigure in Fig 1 shows the evolution of $e(t)$ in some simulation cases. It is obvious that $e(0) = 0.01$ and in some cases, the values of $e(t)$ increase and reach a maximum in the first 10 hours, then decrease asymptotically. To evaluate the convergence performance, the histogram of the maximum value of state error in each case is plotted in the right subfigure in Fig 1. The maximum state errors in 99.99% simulation cases are less than or equal to 0.04, which corresponds to about 40 minutes in the circadian phase shift and is small enough to be ignored during entrainment. Based on Hoeffding inequality [1], the probability of $\max(e(t)) \leq 0.04$ is greater than 98.47% with a confidence level of 99%. These results imply that in most cases the entraining state remains consistent with the reference state under reference light and spontaneous sleep schedule after reaching the terminal condition (0.3) with tol=0.01.

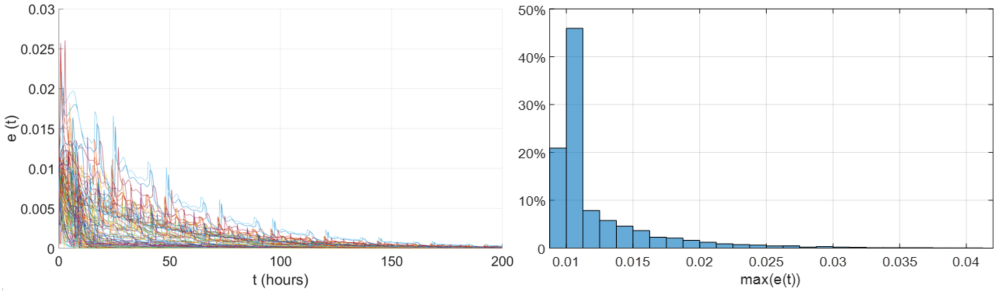

Fig 1. The time evolution of state error $e(t)$ with initial values $[x(0), x_c(0), H(0)]$ satisfying (0.3) and $n(0)$ valued between 0 and 1 (left), and the histogram of maximum value of $e(t)$ in each case (right).

0.2 Optimal light input for minimum-time entrainment with spontaneous sleep

Assume the mode transition between the ith and $(i+1)$th modes occurs at time $t = t_i$, with the state following the switching condition, which is expressed as a function of state and switching time t_i given as

$$\varphi_i(y(t_i), t_i) = 0. \tag{0.4}$$

The entrainment process terminates at the final time of the Nth mode $t_f = t_N$ with a terminal condition defined as

$$\varphi_N(y(t_N), t_N) = \varphi_f(y(t_f), t_f) = 0. \tag{0.5}$$

Notation: Denote $y(t_i)$ and $I(t_i)$ as y_i and I_i, respectively, and represent the partial derivatives in the following forms:

$$\varphi_{i,y}(y,t) \triangleq \frac{\partial \varphi_i(y,t)}{\partial y},\ \varphi_{i,t}(y,t) \triangleq \frac{\partial \varphi_i(y,t)}{\partial t},\ F_{i,y}(y,I) \triangleq \frac{\partial F_i(y,I)}{\partial y},\ F_{i,I}(y,I) \triangleq \frac{\partial F_i(y,I)}{\partial I},$$

$$L_y(t,y,I) \triangleq \frac{\partial L(t,y,I)}{\partial y},\ L_I(t,y,I) \triangleq \frac{\partial L(t,y,I)}{\partial I}.$$

Under the dynamics in Eq (0.1), the state y is only decided by input $I(t)$ with a fixed initial condition. Given switching and terminal conditions in Eq (0.4), (0.5) and a fixed

y_0, the switching and final time $t_1, ..., t_N$ are also completely determined by $I(t)$. To take the switching/terminal conditions and dynamics equation into account, we introduce multipliers $P(t)$ and ϵ_i, $i \in [1, ..., N]$, where $P(t)$ is a function of time and ϵ_i ($i \in [1, ..., N]$) are scalars. The augmented cost is written as:

$$J_a(I; y_0) = \int_{t_0}^{t_N} L(\tau, y(\tau), I(\tau))d\tau$$
$$+\sum_{i=1}^{N} \int_{t_{i-1}}^{t_i} P^T(\tau)\left[F_i(y(\tau), I(\tau)) - \dot{y}(\tau)\right] d\tau + \sum_{i=1}^{N} \epsilon_i \varphi_i(y_i, t_i). \quad (0.6)$$

The value of J_a in Eq (0.6) is equal to the value of J in Eq (0.2) as Eq (0.1), (0.4) and (0.5) are satisfied during entrainment. For a feasible choice of light input $I(t)$ and the corresponding state $y(t)$ and switching times $t_1, ..., t_N$, the input $I(t)$ is perturbed as $I(t) + \alpha\xi(t) \in \Omega_I$ with a scalar α which is sufficiently close to 0 and an admissible perturbation term $\xi(t)$. The state and the switching/final times resulting from the perturbed input are given as $y(t) + \alpha\eta(t) + o(\alpha)$ and $t_i + \alpha\sigma_i + o(\alpha)$ respectively, where $o(\alpha)$ represents a higher order term of α, $\eta(t)$ and σ_i represent the first-order variation of $y(t)$ and t_i with respect to $\xi(t)$, i.e.,

$$\eta(t) = \lim_{\alpha\to 0} \frac{d}{d\alpha}[y(I + \alpha\xi, t) - y(I, t)],$$
$$\sigma_i = \lim_{\alpha\to 0} \frac{d}{d\alpha}[t_i(I + \alpha\xi) - t_i(I)].$$

The perturbed augmented cost is demonstrated as

$$J_a(I + \alpha\xi; y_0) = \int_{t_0}^{t_N+\alpha\sigma_N+o(\alpha)} L(\tau, y + \alpha\eta + o(\alpha), I + \alpha\xi)d\tau$$
$$+\sum_{n=1}^{N} \int_{t_{i-1}+\alpha\sigma_{i-1}+o(\alpha)}^{t_i+\alpha\sigma_i+o(\alpha)} P^T(\tau)[F_i(y + \alpha\eta + o(\alpha), I + \alpha\xi) - \dot{y} - \alpha\dot{\eta} - o(\alpha)]d\tau$$
$$+\sum_{i=1}^{N} \epsilon_i\varphi_i(y_i + \alpha\eta_i + \alpha F_i(y_i + \alpha\eta_i + o(\alpha), I + \alpha\xi)\sigma_i + o(\alpha), t_i + \alpha\sigma_i + o(\alpha)),$$

where $\sigma_0 = 0$. The first-order variation of the augmented cost is calculated by

$$
\begin{aligned}
\delta J_a|_I =& \lim_{\alpha\to 0}\frac{d}{d\alpha}[J_a(I+\alpha\xi;y_0)-J_a(I;y_0)] \\
=& \int_{t_0}^{t_N} L_y^T(\tau,y,I)\eta + L_I(\tau,y,I)\xi d\tau \\
&+\sum_{i=1}^{N}\int_{t_{i-1}}^{t_i}\dot{P}^T\eta + P^T[F_{i,y}^T(y,I)\eta + F_{i,I}(y,I)\xi]d\tau \\
&+\sigma_N\left[L(t_N,y_N,I_N)+P_N^T(F_N(y_N,I_N)-\dot{y}_N)\right] \\
&+\sum_{i=1}^{N}\epsilon_i[\varphi_{i,y}^T(y_i,t_i)(\eta_i+F_i(y_i,I_i)\sigma_i)+\varphi_{i,t}(y_i,t_i)\sigma_i] \\
&+\sum_{i=1}^{N-1}[P^T(t_i^-)F_i(y_i,I_i)-P^T(t_i^+)F_{i+1}(y_i,I_i)]\sigma_i \\
&-\sum_{i=1}^{N}\left[P^T(t_i^-)\eta(t_i)-P^T(t_{i-1}^+)\eta(t_{i-1})\right],
\end{aligned}
$$

where t_i^- and t_i^+ denote the times just before and after the switching condition (21) occurs, $\eta(t_0)=0$ for given initial condition. For the algorithm that we present in this section, it suffices to know that if we choose their values such that

$$
\frac{dP(t)}{dt} = - F_{i,y}(y,I)P(t) - L_y(t,y,I) \text{ when } t\in[t_{i-1},t_i), \tag{0.7a}
$$

$$
P(t_N) = \epsilon_N\varphi_{N,y}(y_N,t_N), \tag{0.7b}
$$

$$
\epsilon_N = -\frac{L(t_N,y_N,I_N)}{\varphi_{N,t}(y_N,t_N)+\varphi_{N,y}^T(y_N,t_N)F_N(y_N,I_N)}, \tag{0.7c}
$$

$$
P(t_i^-) = \epsilon_i\varphi_{i,y}(y_i,t_i)+P(t_i^+), \tag{0.7d}
$$

$$
\epsilon_i = -\frac{P^T(t_i^-)F_i(y_i,I_i)-P^T(t_i^+)F_{i+1}(y_i,I_i)}{\varphi_{i,y}^T(y_i,t_i)F_i(y_i,I_i)+\varphi_{i,t}(y_i,t_i)}, \tag{0.7e}
$$

the variation in the augmented cost function is simplified as

$$
\delta J_a|_I = \int_{t_0}^{t_N}[L_I(\tau,y,I)+P^T(\tau)F_I(y,I)]\xi(\tau)d\tau.
$$

Therefore, the gradient of the augmented cost to the input I is expressed in the following form:

$$
\nabla_{I(t)}J_a = L_I(t,y,I)+P^T(t)F_{i,I}(y,I). \tag{0.8}
$$

From Eq (0.7d) we know that the value of $P(t)$ is discontinuous at the switching time. For the S+C3 model, if the subject falls asleep spontaneously at t_i, the state satisfies

$$
\varphi_i(y_i,t_i) = H_i - A_c x_i - 0.67 = 0,
$$

while if the subject wakes up spontaneously at t_i, the state follows

$$
\varphi_i(y_i,t_i) = H_i - A_c x_i - 0.17 = 0.
$$

Plugging these equations and the state equation into (0.7d) and (0.7e), we determine the value of $P(t_i^-)$ based on $P(t_i^+)$ and y_i.

Steps of a gradient descent process for solving the optimal light input for minimum-time entrainment with the spontaneous sleep schedule are listed as below:

1. Denote the light, state, $P(t)$, t_i and t_f in the hth iteration as $I^h(t)$, $y^h(t)$, $P^h(t)$, t_i^h and t_f^h, respectively. Set $h = 0$ and choose an initial guess of the light input as $I^0(t)$;

2. Integrate the state equation forward, determine the switching time $t_1^h, ..., t_{N-1}^h$ and switching state $y_1^h, ..., y_{N-1}^h$, final time t_f^h and the final state $y^h(t_f^h)$ and $y_{\text{ref}}(t_f^h)$;

3. Determine the final value of $P^h(t_f^h)$ based on Eq (0.7b), (0.7c) and simulate (0.7a) backward in every mode. At the sleep time and wake time points, $P^h(t_i^{h-})$ is decided based on Eq (0.7d) and (0.7e);

4. Determine the gradient of the augmented cost with respect to $I^h(t)$ based on Eq (0.8), update I^h by

$$I^{h+1}(t) = \min\left\{\max\left[I^h(t) - \eta_I \nabla_{I^h(t)} J_a, 0\right], I_{\max}\right\}, \tag{0.9}$$

where $\eta_I > 0$ is the updating step for $I(t)$. It is determined by a line search.

5. Set $h = h + 1$ and repeat the steps 2-5 until the solution converges to a stable value.

Lemma 1 *(From [2]) If the iteration reaches a fixed point, i.e., $I^*(t) = I^{h+1}(t) = I^h(t)$ for any positive step size η_I, the following condition must be satisfied for all $t \in [t_0, t_f^h]$*

$$\begin{cases} I^h(t) = 0 & \text{when } \nabla_{I^h(t)} J_a > 0, \\ I^h(t) = I_{\max} & \text{when } \nabla_{I^h(t)} J_a < 0, \\ I^h(t) \in [0, I_{\max}] & \text{when } \nabla_{I^h(t)} J_a = 0. \end{cases} \tag{0.10}$$

If the final optimal solution is valued at either maximum or minimum, i.e., $I^*(t) \in \{0, I_{\max}\}$ for $\forall\ t \in [t_0, t_f]$, this control is called the *bang-off control.* If $\nabla_{I(t)} J = 0$ in a time interval, the light input $I(t)$ cannot be updated by the gradient descent process and could be any value between 0 and $I_{\max}$ in this interval. We call this interval the *singular region.*

0.3 Optimal light input and sleep schedule for minimum-time entrainment with controllable sleep

In this part, the sleep schedule is partially controllable during entrainment. Assume that the sleep-wake switching time t_i, $\forall i \in [1, ..., N-1]$ follows inequality constraints given in the following form:

$$\varphi_{i\min} \le \varphi_i(y_i, t_i) \le \varphi_{i\max},\ \forall i \in \{1, ..., N-1\}, \tag{0.11}$$

where $\varphi_{i\min}$ and $\varphi_{i\max}$ are the lower and upper bounds of φ_i. The augmented cost function with controllable sleep is written as

$$J_a(I, t_1, ..., t_{N-1}; y_0) = \int_{t_0}^{t_N} L(\tau, y(\tau), I(\tau)) d\tau$$

$$+ \sum_{i=1}^{N} \int_{t_{i-1}}^{t_i} P^T(\tau) \left[F_i(y(\tau), I(\tau)) - \dot{y}(\tau)\right] d\tau + \epsilon_N \varphi_N(y_N, t_N).$$

For the entrainment problem with controllable sleep schedule and a fixed initial condition in Section 3.3, the entrainment time and state are fully determined by the switching times and light input. To evaluate the effects of both light and sleep schedule on the entrainment time, for a feasible choice of $(I(t), t_1, ..., t_{N-1})$, we perturb the light input and switching times as $I(t) + \alpha\xi(t) \in \Omega_I$ and $t_i + \alpha\sigma_i$, $i \in [1, ..., N-1]$, following

Eq (0.11), with a scalar α sufficiently close to 0 and admissible perturbation terms $\xi(t)$ and σ_i. The perturbed augmented cost is given as

$$
\begin{aligned}
&J_a(I+\alpha\xi, t_1+\alpha\sigma_1, ..., t_{N-1}+\alpha\sigma_{N-1}; y_0)\\
&= \int_{t_0}^{t_N+\alpha\sigma_N+o(\alpha)} L(\tau, y+\alpha\eta+o(\alpha), I+\alpha\xi)d\tau\\
&+\sum_{i=1}^{N}\int_{t_{i-1}+\alpha\sigma_{i-1}}^{t_i+\alpha\sigma_i} P^T(\tau)[F_i(y+\alpha\eta+o(\alpha), I+\alpha\xi)-\dot{y}-\alpha\dot{\eta}-o(\alpha)]d\tau\\
&+\epsilon_N\varphi_N(y_N+\alpha\eta_N+\alpha F_N(y_N+\alpha\eta_N, I+\alpha\xi)\sigma_N+o(\alpha), t_N+\alpha\sigma_N+o(\alpha)).
\end{aligned}
$$

The first-order variation of the augmented cost is

$$
\begin{aligned}
\delta J_a|_{I,t_1,...,t_{N-1}} = &\int_{t_0}^{t_N} L_y^T(\tau, y, I)\eta + L_I(\tau, y, I)\xi d\tau\\
&+\sum_{i=1}^{N}\int_{t_{i-1}}^{t_i}\dot{P}^T\eta + P^T(\tau)[F_{i,y}^T(y, I)\eta + F_{i,I}(y, I)\xi]d\tau\\
&-\sum_{i=1}^{N}[P^T(t_i^-)\eta(t_i) - P^T(t_{i-1}^+)\eta(t_{i-1})]\\
&+\epsilon_N\{\varphi_{N,y}^T(y_N, t_N)[\eta_N + F_N(y_N, I_N)\sigma_N] + \varphi_{N,t}(y_N, t_N)\sigma_N\}\\
&+\sum_{i=1}^{N-1}[P^T(t_i^-)F_i(y_i, I_i) - P^T(t_i^+)F_{i+1}(y_i, I_i)]\sigma_i\\
&+\sigma_N L(t_N, y_N, I_N).
\end{aligned}
$$

We choose the values of $P(t)$ and ϵ_N in the following forms:

$$
\begin{aligned}
P(t_N) =&\epsilon_N\varphi_{N,y}(y_N, t_N), && (0.12a)\\
\epsilon_N =& -\frac{L(t_N, y_N, I_N)}{\varphi_{N,y}^T(y_N, t_N)F_N(y_N, I_N) + \varphi_{N,t}(y_N, t_N)}, && (0.12b)\\
P(t_i^-) =&P(t_i^+), && (0.12c)\\
\frac{dP(t)}{dt} =& -F_{i,y}(y, I)P(t) - L_y(t, y, I) \text{ when } t\in[t_{i-1}, t_i). && (0.12d)
\end{aligned}
$$

After some simplifications, the variation of the augmented cost function is given as

$$
\begin{aligned}
\delta J_a|_{I,t_1,...,t_{N-1}} = &\int_{t_0}^{t_N}\left[L_I(\tau, y, I) + P^T(\tau)F_I(y, I)\right]\xi d\tau\\
&+\sum_{i=1}^{N-1} P^T(t_i)[F_i(y_i, I_i) - F_{i+1}(y_i, I_i)]\sigma_i.
\end{aligned}
$$

The equation above provides the variation of augmented cost function resulting from a small perturbation in light input and switching times. The gradients of the augmented cost to I and t_i are given as

$$
\begin{aligned}
\nabla_{I(t)}J_a =&L_I(t, y, I) + P^T(t)F_{i,I}(y, I), && (0.13a)\\
\nabla_{t_i}J_a =&P^T(t_i)[F_i(y_i, I_i) - F_{i+1}(y_i, I_i))]. && (0.13b)
\end{aligned}
$$

Unlike the case in the minimum-time entrainment with spontaneous sleep, here $P(t)$ is continuous at the switching points in (0.12c). Based on the gradient descent method, if we represent the ith switching time at the hth iteration as t_i^h, its value is updated by:

$$t_i^{h+1} = t_i^h - \eta_{\text{switch}} \nabla_{t_i^h} J, \tag{0.14}$$

where η_{switch} is the updating step for the switching time and also determined by a line search in the simulation that guarantees that t_i satisfies the constraint in Eq (0.11) in every iteration. Steps of calculation of the optimal light and sleep schedule for minimum-time entrainment are listed below:

1. Denote the light, state, $P(t)$, $\beta(t)$, t_i and t_f in the hth iteration as $I^h(t)$, $y^h(t)$, $P^h(t)$, $\beta^h(t)$, t_i^h and t_f^h, respectively. Set $h = 0$ and choose an initial guess of the light input $I^0(t)$ and the sleep schedule $\beta^0(t)$ to drive the two-process state to the reference trajectory in a finite time;

2. Integrate the state equation forward, determine the switching time $t_1^h, ..., t_{N-1}^h$, switching state $y_1^h, ..., y_{N-1}^h$, final time t_f^h and the final states $y^h(t_f^h)$ and $y_{\text{ref}}(t_f^h)$;

3. Determine the final value of $P^h(t_f^h)$ based on Eq (0.12a), (0.12b) and integrate (0.12d) backward to get $P^h(t)$, $t \in [t_0, t_f^h]$;

4. Determine the gradient of the augmented cost to $I^h(t)$ and the sleep/wake times based on (0.13), update I^h, t_i^h based on Eq (0.9) and (0.14);

5. Set $h = h + 1$ and repeat the steps 2-5 until both $I^h(t)$ and $\beta^h(t)$ converge to some stable solutions.

Note that the final solution of $I(t)$ from this gradient descent process still holds the condition in Eq (0.10).

References

1. Hoeffding W. Probability inequalities for sums of bounded random variables. J Am Stat Assoc. 1963;58(301):13-30.

2. Julius AA, Yin J, Wen JT. Time optimal entrainment control for circadian rhythm. PLoS One. 2019 Dec 18;14(12):e0225988.
